# Phylogeny of the Madagascar-centred tribe Danaideae (Rubiaceae) as a precursor to taxonomic revision: insights into its generic and species limits, affinities and distribution

**DOI:** 10.1093/aob/mcac121

**Published:** 2022-09-23

**Authors:** Sylvain G Razafimandimbison, Niklas Wikström, Anbar Khodabandeh, Catarina Rydin

**Affiliations:** Department of Botany, Swedish Museum of Natural History, Box 50007, SE-104 05 Stockholm, Sweden; Department of Ecology, Environment and Plant Sciences, Stockholm University, SE-106 91 Stockholm, Sweden; The Bergius Foundation, The Royal Swedish Academy of Sciences, SE-104 05 Stockholm, Sweden; Department of Ecology, Environment and Plant Sciences, Stockholm University, SE-106 91 Stockholm, Sweden; The Bergius Foundation, The Royal Swedish Academy of Sciences, SE-104 05 Stockholm, Sweden; Department of Ecology, Environment and Plant Sciences, Stockholm University, SE-106 91 Stockholm, Sweden; The Bergius Foundation, The Royal Swedish Academy of Sciences, SE-104 05 Stockholm, Sweden

**Keywords:** Danaideae, *Danais*, Madagascar, morphology, *Payera*, phylogeny, Rubiaceae, *Schismatoclada*, systematics, taxonomy, Western Indian Ocean Region

## Abstract

**Background and aims:**

The tribe Danaideae (Rubiaceae) is almost exclusively endemic to the Western Indian Ocean Region (WIOR), and encompasses the genera *Danais*, *Payera* and *Schismatoclada* that occur in humid, sub-humid and mountain and mountain bio climate zones. Much of the species diversity is endemic to restricted, remote and/or mountainous areas of Madagascar and recent field work on the island indicates substantial unknown diversity of the Danaideae. Furthermore, the monophyly of the Malagasy genera *Payera* and *Schismatoclada* has been questioned in previous work, species delimitations and phylogenetic relationships within the genera are poorly understood, and the distribution and evolution of gross morphological features have not been assessed.

**Methods:**

We conducted morphological investigations, and produced robust phylogenies of Danaideae based on nuclear and plastid sequence data from 193 terminals. Ample plant material has been newly collected in the WIOR for the purpose of the present study, including potentially new species unknown to science. We performed Bayesian non-clock and relaxed-clock analyses employing three alternative clock models of a dataset with a dense sample of taxa from the entire geographical ranges of Danaideae. Based on the results, we discuss species diversity and distribution, relationships, and morphology in Danaideae.

**Key results:**

Our results demonstrate the monophyly of Danaideae, its three genera and 42 species. Nine species are resolved as non-monophyletic. Many geographically distinct but morphologically heterogeneous lineages were identified, and morphological features traditionally considered diagnostic of subgroups of the genera, used for example in species identification keys, are not clade-specific.

**Conclusions:**

Our results demonstrate that Madagascar contains ample previously undocumented morphological and species diversity of Danaideae. Our novel approach to molecular phylogenetic analyses as a precursor to taxonomic revisions provides numerous benefits for the latter. There are tentative indications of parallel northward diversification in *Payera* and *Schismatoclada* on Madagascar, and of geographical phylogenetic clustering despite the anemochorous condition of Danaideae.

## INTRODUCTION

**Fig. 1.**
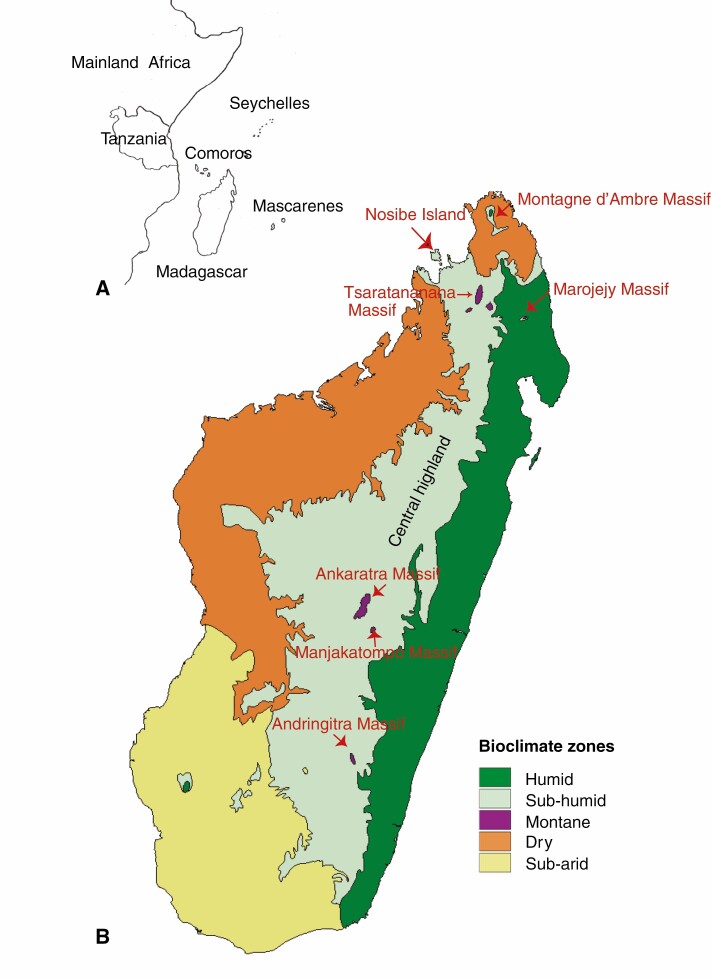
The distribution of Danaideae. The genus *Danais* has a broad distribution in the Western Indian Ocean Region (WIOR), extending to Tanzania of mainland Africa (A). *Payera* and *Schismatoclada* are restricted to Madagascar, where all three genera occur in the humid, sub-humid and mountain bioclimatic zones of the island (B). Map modified from Rakotoarivelo *et al.* (2019).

The Western Indian Ocean Region (WIOR) is one of the world’s biodiversity hotspots, encompassing Madagascar and the neighbouring Comoros, Mascarene and Seychelles archipelagos (Myers *et al.*, 2000) (Fig. 1). Madagascar is by far the largest island (~593 000 km^2^) in the region and its extant biota mostly encompasses the descendants of Cenozoic dispersers, often from mainland Africa (e.g. Yoder and Nowak, 2006), with few groups with Asian, Neotropical and Pacific origins (e.g. Rouhan *et al.*, 2012; Razafimandimbison *et al.*, 2017). Madagascar has a complex landscape dominated by mountainous areas running north–south, stretching from the Tsaratanana Massif and its satellites in the north to the Anosy mountain chains in the southeast, and resulting in a central highland above 800 m (Fig. 1). The eastern slope of the central highland is narrow and falls steeply towards the Indian Ocean. By contrast, its western slope is occupied by a large plain declining progressively towards the Mozambique Channel (e.g. Tattersall and Sussman, 1975). The island has five distinct bioclimatic zones and diverse vegetation types, partly due to the impacts of the southeastern trade winds (Alizé) and the north-western monsoon from the Equator (Koechlin, 1972; Koechlin *et al.*, 1974; Jury, 2003; Fig. 1). The eastern region (0–800 m in altitude) is humid, and harbours littoral forests (at sea level and on sandy soils) and lowland rainforests (including a narrow strip of rainforests along the east coast and the low-elevation rainforests from sea level up to 800 m). The central highland (800–2000 m in altitude) is sub-humid, and hosts montane (or highland) rainforests, sclerophyllous montane forests, woodland savannahs and ericoid thickets. These highland rainforests, woodland savannahs and sclerophyllous forests are intermixed, while the ericoid thickets are characteristics of the high (above 2000 m in altitude), isolated mountains of the Andringitra, Ankaratra, Marojejy and Tsaratanana Massifs and their respective satellites. Most of the western region and the north tip (except the Montagne d’Ambre Massif) of Madagascar are dry, harbouring deciduous, dry forests and woodland savannahs; part of northwestern Madagascar (including the Nosibe Island) is sub-humid, hosting semi-deciduous forests. The south and southwestern regions are sub-arid, hosting spiny thickets (Koechlin, 1972; Koechlin *et al.*, 1974; Jury, 2003; Fig. 1).

**Fig. 2.**
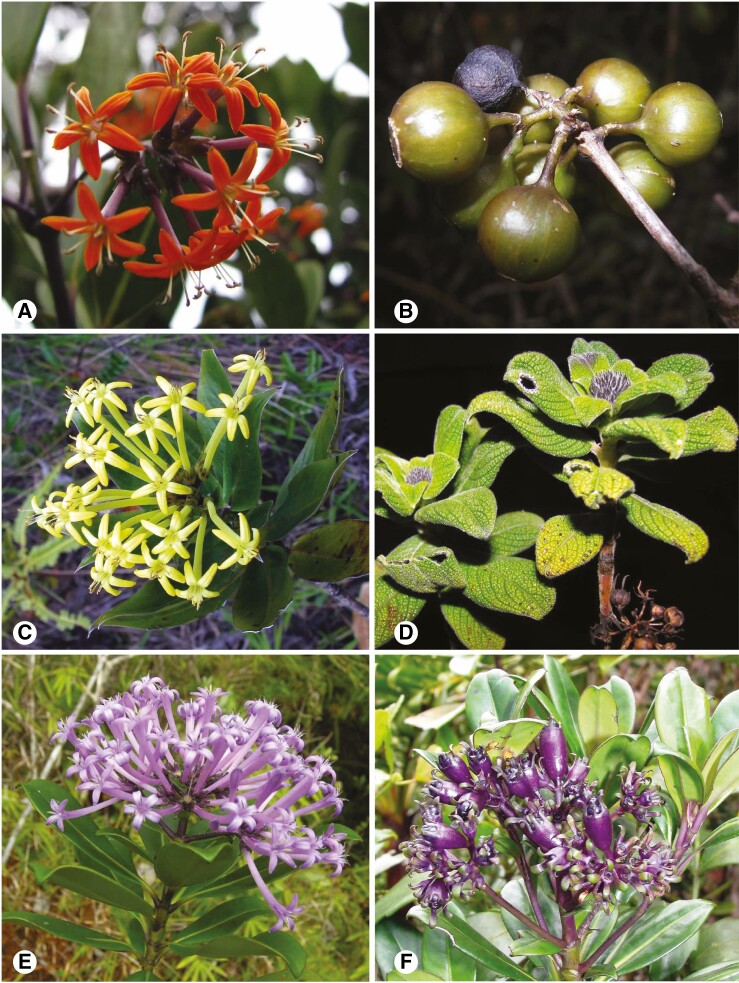
Examples of morphological diversity in the tribe Danaideae. (A) *Danais* ‘*marojejyensis*’ ined.; (B) *Danais pauciflora* Baker; (C) *Payera coriacea* (Humbert) R. Buchner & Puff; (D) *Payera marojejyensis* R. Buchner & Puff; (E, F) *Schismatoclada* ‘*violacea*’ ined. Photo credits. A, D: Kent Kainulainen; B: Patrice Antilahimena, MBG-Mada CC-BY-NC-ND © 2022; and C, E, F: Sylvain G. Razafimandimbison.

Madagascar harbours about 11 400 endemic species, 1743 native genera and 253 families of vascular plants (as of November 2020, Tropicos database). The coffee family (Rubiaceae) is the second largest flowering plant family after the orchid family, with at least 1000 species in 95 genera and 27 tribes on the island. It thus encompasses about 11 % of the land plant diversity of Madagascar, with the level of specific endemism nearly 100 % (Razafimandimbison *et al.*, 2022). Alberteae *sensu*Kainulainen *et al.* (2009) (subfamily Cinchonoideae *sensu lato*) and Danaideae *sensu*Bremer and Manen (2000) (subfamily Rubioideae) are the only two rubiaceous tribes with their species diversity centred in the WIOR. Danaideae (Fig. 2) contain a high proportion of microendemics (endemic species with very small areas of occurrence; Ganzhorn *et al.*, 2014) (Buchner and Puff, 1993; Puff and Buchner, 1994). On Madagascar, the eastern lowland and central highland rainforests of the humid and sub-humid zones (Fig. 1) are the primary habitats of Danaideae, and its geographical range extends from the island’s southeast tip to the northernmost tip (the Montagne d’Ambre Massif) (Fig. 1). Danaideae do not occur in the dry habitats, meaning that its members are entirely absent in the dry and sub-arid zones in western and southwestern Madagascar, respectively (e.g. Buchner and Puff, 1993; Puff and Buchner, 1994; Fig. 1); their centre of species richness is the highland rainforests above 800 m, with only few species found in the eastern littoral and lowland forests, the north-western semi-deciduous forests (from sea level to 800 m), and the ericoid thickets above 2000 m elevation.

Danaideae contain 69 currently accepted species (Supplementary Data Table S1) classified in three genera: the lianescent *Danais* Comm. ex Vent (Fig. 2A, B) (38 species), and the two arborescent (shrubs or small trees) genera *Payera* Baill. (Fig. 2C, D) (10 species) and *Schismatoclada* Baker (Fig. 2E, F) (21 species). *Danais* is widely distributed in the WIOR (but absent in the Seychelles), with most species endemic to Madagascar (Puff and Buchner, 1994; Taylor and Rodgers, 2013), but two occur in the Comoros (one endemic and one species shared with Madagascar; Puff and Buchner, 1994), three are endemic species in the Mascarenes (Verdcourt, 1989) and one species is endemic to mainland Africa (Tanzania; Verdcourt, 1976). By contrast, *Payera* and *Schismatoclada* are Malagasy endemics.

*Danais* (Fig. 2A, B) was originally described by Ventenat (1799), but it was Persoon (1805) who validly published the first *Danais* species, *Danais fragrans* (Comm. ex Lam.) Pers. from the Mascarenes and Madagascar, and described *D. sulcata* Pers. from Mauritius. Specimens of *D. fragrans* from Madagascar and from the Mascarenes (Mauritius and Reunion Islands) have, however, been shown to be distantly related (Krüger *et al.*, 2012), with the latter species forming a clade with the Mauritius *D. sulcata*. As a result, *D. fragrans* is now restricted to the Mascarenes, and the Malagasy *D. lyallii* Baker was resurrected to accommodate the Malagasy *D. fragrans* (Krüger *et al.*, 2012). The first comprehensive morphological study on *Danais* and its allied genera *Payera* and *Schismatoclada* was conducted by Buchner and Puff (1993), who demonstrated their close affinities and proposed new generic circumscriptions. The authors rejected *Danais* as defined by Cavaco (1966), and transferred four arborescent Malagasy species (*Danais bakeriana* Homolle, *D. decaryi* Homolle, *D. madagascariensis* Cavaco and *D. mandrarensis* Homolle ex Cavaco) to *Payera* (Buchner and Puff, 1993), rendering *Danais* an exclusively lianescent group. While this taxonomic adjustment has yet to be assessed using molecular data, *Danais* as delimited by Puff and Buchner (1994) has been widely accepted by the Rubiaceae community. In their revision of the Malagasy and Comorian *Danais*, Puff and Buchner (1994) recognized a total of 26 species (plus two that were considered poorly known). Krüger *et al.*’s (2012) resurrection of *D. lyallii* and six new species from Madagascar described by Taylor and Rogers (2013) bring the total number of Malagasy species of *Danais* to 35. Two additional species, *D. corymbosa* Balf. F. and *D. sulcata*, were treated in the Mascarene Flora of Rubiaceae (Verdcourt, 1989), and the Tanzanian *D. xanthorrhoea* (K. Schum.) Bremek. was included in the East African Flora of Rubiaceae (Verdcourt, 1976), bringing a total of 38 currently accepted species of *Danais* (Supplementary Data Table S1).

The Malagasy genus *Payera* (Fig. 2C, D) was originally described by Baillon (1878) based on the holotype and the sole isotype (both at herbarium P) of *Payera conspicua* Baill. The type species *P. conspicua* may have been an extremely rare species that is now extinct; it is known only from the type specimens collected in 1841. The type locality on Madagascar is unknown and the species has not been recollected for 180 years despite recent efforts. As a result, the genus remained virtually unknown until Buchner and Puff (1993) conducted their detailed morphological study on the so-called ‘genus complex *Danais*–*Schismatoclada–Payera*’ (now Danaideae *sensu*Bremer and Manen, 2000), including the Malagasy monospecific genus *Coursiana* Homolle (Homolle, 1942). They report that the former director of the Paris herbarium, Professor J.-F. Leroy, based on his unpublished notes, was the first to have postulated close affinities of *Payera* with *Danais*, *Coursiana* and *Schismatoclada*. Leroy’s hypothesis was endorsed by Buchner and Puff (1993), who used corolla pubescence, corolla aestivation type and fruit dehiscence as primary characters to recircumscribe the two arborescent Danaideae genera (*Payera* and *Schismatoclada*). As a consequence, Buchner and Puff (1993) formally transferred two dwarf or low-growing shrubby species of *Schismatoclada* (*S. beondrokensis* Humbert and *S. coriacea* Humbert, Humbert, 1955) endemic to the Marojejy Massif in northeastern Madagascar (Fig. 1) to *Payera*. Furthermore, Buchner and Puff (1993) formally merged the Malagasy monotypic genus *Coursiana* in *Payera*, and described two new species of *Payera* (*P. glabrifolia* J.-F. Leroy ex R. Buchner and Puff and *P. marojejyensis* R. Buchner and Puff). These taxonomic adjustments [together with Buchner’s and Puff’s (1993) transfer of the four arborescent *Danais* species to *Payera*] increased the total number of *Payera* species to 10 (Supplementary Data Table S1).

The Malagasy genus *Schismatoclada* (Fig. 2E, F) was originally described by Baker (1883) to accommodate *Schismatoclada psychotrioides* Baker. The genus is characterized by its capsular, elongated and conspicuously beaked fruits (Buchner and Puff, 1993; Fig. 2F). Three more species, *S. concinna* Baker, *S. viburnoides* Baker and *S. tricholarynx* Baker, were subsequently added (Baker, 1885, 1887). Homolle (1939) studied the specimens of *Schismatoclada* at the P herbarium, and described ten new species. Homolle also transferred to *Schismatoclada* two species from the genus *Mussaenda* L. (Rubiaceae), *S. citrifolia* (Lam. ex Poir.) Homolle and *S. thouarsiana* (Baill.) Homolle, and merged Baker’s *S. tricholarynx* with *S. psychotrioides*. Subsequently, Boiteau (1941) described *Schismatoclada homolleae* Boiteau (as *S. homollei*). Humbert (1955) then described three additional species, *S. beondrokensis* [now *Payera beondrokensis* (Humbert) R. Buchner and Puff], *S. coriacea* [now *P. coriacea* (Humbert) R. Buchner and Puff] and *S. marojejyensis* Humbert from the Marojejy Massif (Fig. 1). Later, Cavaco (1964, 1967, 1968) studied the *Schismatoclada* specimens at P herbarium, and accepted all known *Schismatoclada* species recognized by Baker (1883, 1885, 1887) and Homolle (1939), with the exception of *S. mandrarensis* Homolle, which he merged with *S. aurantiaca* Homolle. Cavaco described four new species, *S. bracteata* Homolle ex Cavaco and *S. villiflora* Homolle ex Cavaco (Cavaco, 1964), *S. longistipula* Cavaco (Cavaco, 1967), and *S. coursiana* Cavaco (Cavaco, 1968). Finally, a new *Schismatoclada* species from the Marojejy Massif, *S. spathulata* Strid and Razafim., was recently described by Strid *et al.* (2019), bringing the total number of species of *Schismatoclada* to 21 (Supplementary Data Table S1).

The monophyly of Danaideae is strongly supported by molecular data (e.g. Bremer and Manen, 2000; Krüger *et al.*, 2012). Beside their isolated geographical range in the WIOR (with the exception of the Tanzanian *Danais xanthorrhoea*), the members of the tribe can be characterized by a combination of the following character states: presence of raphides in tissues, heterodistylous flowers, valvate corolla aestivation, dry capsular fruits and winged seeds (Buchner and Puff, 1993). Features such as growth habit, aestivation of corolla lobes, corolla pubescence and fruit type have been used for genus recognition in Danaideae (Buchner and Puff, 1993), but the plesiomorphic or apomorphic status of these features, i.e. their evolutionary history, have not been tested. For example, while *Danais* and *Schismatoclada* are more similar to each other than either is to *Payera* regarding the large bracts subtending the inflorescences (mostly absent vs. mostly present), aestivation of corolla lobes (valvate-reduplicate vs. valvate *s.s.*), and corolla pubescence (mostly absent vs. covered by appressed, silvery hairs) (e.g. Buchner and Puff, 1993; Schatz, 2001), *Payera* and *Schismatoclada* share an arborescent habit and capsular fruits that are conspicuously beaked (Fig. 2D, F), while *Danais* and *Payera* have capsular fruits that dehisce loculicidally (Buchner and Puff 1993: 62). Molecular phylogenetics is an important tool that allows systematists to assess the phylogenetic value of such characters.

The first molecular phylogenetic study that focused on Danaideae (Krüger *et al.*, 2012) indicated that *Payera* and *Schismatoclada* as defined by Buchner and Puff (1993) are mutually paraphyletic, and thus untenable, but the authors refrained from making any taxonomic adjustment, because the type of *Payera* (*P. conspicua*) was not investigated and statistical support for results were partly weak. Furthermore, the sampling of *Payera* and *Schismatoclada* available to Krüger and colleagues at the time (2012) did not cover the entire geographical ranges of these genera. For example, none of the *Schismatoclada* species from southeastern Madagascar and the *Payera* species from central east of the island was included in the study, and many included specimens were not identified, or were misidentified, at the genus and/or species level. In association with ongoing taxonomic revisions, we have re-identified all the Danaideae specimens included in Krüger *et al.* (2012), and ample newly collected plant material is available from subsequent recent field work by us. It is thus timely and highly relevant to conduct a new phylogenetic study of Danaideae.

The main objective of the present study is to investigate species diversity and phylogenetic relationships in the tribe Danaideae based on morphology and molecular data. We included 193 samples, many of which are newly collected in remote areas of Madagascar representing 43 accepted species and 37 (potentially) new yet undescribed species of Danaideae identified during ongoing taxonomic revisions (Supplementary Data Tables S1 and S2). The resulting phylogenies are subsequently utilized to: (1) re-assess the monophyly of the three genera of Danaideae, (2) investigate species delimitations and relationships within the tribe, and (3) evaluate the distribution and evolution of gross morphological characters commonly used in floras and taxonomic work of Danaideae.

## MATERIALS AND METHODS

### Taxon sampling

We investigated as many Danaideae species as possible for the present study, including those analysed in Krüger *et al.* (2012) and a substantial set of new plant material collected by us for the purpose of the present study during field work over the entire distribution area of the tribe. Most of the sampled species were represented by two–three individuals, while species known to be morphologically variable with wide geographical distributions (e.g. *Danais cernua* Baker, *D. lyallii*, *Schismatoclada concinna*, *S. farahimpensis* Homolle and *S. psychotrioides*) were represented by many individuals. We were not able to obtain sequenceable material for all species of Danaideae (i.e. species listed in grey in Supplementary Data Table S1). On the other hand, a total of 37 as yet undescribed and potentially new species of *Danais*, *Payera* and *Schismatoclada* were included in our study. Eight of the 31 included species of *Danais*, three of the 11 included species of *Payera* and 19 of the 31 included species of *Schismatoclada* are potentially new yet undescribed species. In addition to these 30 new and 43 currently accepted species, which were all investigated using morphology and molecular data, two potentially new species of *Payera* and five new species of *Schismatoclada* (for which no molecular data were produced) were studied morphologically. Six species from six tribes of the Spermacoceae alliance and seven species from seven tribes of the Psychotrieae alliance were included as outgroups. The species from the Psychotrieae alliance were used to root the trees. Of the total 193 analysed samples, 74 were from *Danais* (representing 23 of the 38 accepted species + eight newly proposed species, ~67 %), 21 samples were from *Payera* (representing eight of the ten accepted species + three of the five newly proposed species, ~73 %) and 85 samples were from *Schismatoclada* (representing 11 of the 21 accepted species + 19 of 24 newly proposed species, ~67 %). Voucher information, country of origin of these samples and sequence accession numbers are specified in Table S2.

### Morphological studies

All sampled Danaideae species included in the present study have been carefully studied using stereomicroscopy and conventional techniques. The specimens were identified with the aid of species keys in Puff and Buchner (1994) and Taylor and Rodgers (2013) for *Danais*, in Buchner and Puff (1993) for *Payera*, and in Cavaco (1964) and Buchner and Puff (1993) for *Schismatoclada.* Scanned images of the type specimens of *Danais*, *Payera* and *Schismatoclada* species available through the databases of the Kew Royal Garden (WCSP, 2022), Missouri Botanical Garden (Tropicos, 2022) and Paris herbarium (MNHN, Chagnoux S, 2022) were accessed for comparison with the studied material, which comprises more than 800 specimens of these genera loaned from the G, BR, K, MO, P, S, UPS, TAN and TEF herbaria (Thiers, 2016). We placed particular emphasis on morphological variation within and among species regarding features used in these floral and classificational works, i.e. leaf size, type and phyllotaxy, stipule type and flower colour. These morphological investigations are in addition part of extended morphological studies conducted by the first author (S.G.R.) for ongoing taxonomic revisions of *Payera* and *Schismatoclada*.

### Choice of molecular markers, molecular laboratory procedures and data assembly

We used three plastid (*ndhF*, *matK* including *trnK*, and *trnT-F*) and nuclear ribosomal ITS (nrITS) markers for our study. DNA extraction and amplification were achieved following the protocols outlined in Razafimandimbison *et al.* (2004) for nrITS, Bremer *et al.* (1999) for *ndhF*, Kainulainen and Bremer (2014) for *matK*, and Razafimandimbison and Bremer (2002) for *trnT-F*. The same primers used for PCRs were utilized for sequencing reactions, which were sent to Macrogen Europe (Amsterdam, the Netherlands) for sequencing. The new sequence data were assembled using the Staden package v.2.0.09b (Staden, 1996). For each marker, sequences were aligned using MUSCLE v.3.8.31 (default settings; Edgar, 2004), as implemented in AliView v.1.18.1 (Larsson, 2014). Manual adjustments were subsequently done for the nrITS and *trnT-F* datasets following the similarity criterion (Simmons, 2004) using AliView.

### Phylogenetic non-clock analyses

Phylogenetic reconstructions were achieved using the Bayesian Markov chain Monte Carlo (MCMC) method (Yang and Rannala, 1997) as implemented in the software MrBayes v.3.2.7b (Ronquist *et al.*, 2012). Data partitioning was selected using the software PartitionFinder v.2 (Lanfear *et al.*, 2017), which indicated a best-fitting partitioning scheme employing two partitions for our data, one comprising *trnT-F* and a second comprising nrITS, *matK* and *ndhF*. The general time reversible substitution model with rate variation across sites modelled as a gamma distribution (GTR+Γ) was used for *trnT-F*. For the partition including the nrITS, *matK* and *ndhF* datasets, the general time reversible substitution model with rate variation across sites modelled as a gamma distribution and including a proportion of invariable sites (GTR+Γ+I) was used. Model selection was based on the corrected Akaike information criterion as calculated utilizing MrAIC v.1.4.6, (Nylander, 2004). Gaps were treated as missing data in all alignments. Inferred indels/deletions were not coded as separate characters, and sites considered ambiguously aligned in the nrITS and *trnT-F* regions were removed from the analyses. Individual gene regions were initially analysed separately using Bayesian inference and substitution models as specified above.

All separate and combined Bayesian analyses were run on the CIPRES computing cluster (Miller *et al.*, 2010). The combined data matrix is provided in nexus format (Supplementary Data File S1). Each analysis comprised two runs of four chains each that were run for 20 million generations, sampling trees and parameters every 2000th generation. For all analyses, stationarity and convergence of runs were checked using the program AWTY (Nylander *et al.*, 2008). The effective sample size (ESS) of parameters was monitored using the program TRACER v.1.6 (Rambaut and Drummond, 2013). Trees sampled before the Bayesian posterior probability (BPP) of splits stabilized were excluded as a burn-in phase. All saved trees from the two independent runs were subsequently pooled for a consensus tree.

### Phylogenetic relaxed-clock analyses

With the primary objective to infer phylogenetic relationships within a relaxed-clock framework (not to estimate node ages), we performed relaxed-clock analyses using three alternative relaxed-clock models, the Brownian motion model (TK02) described by Thorne and Kishino (2002), the white noise model (WN) described by LePage *et al.* (2007) and the independent lognormal model (ILN) described by Drummond *et al.* (2006). The fossil *Morinda chinensis* from the middle Eocene of south China (Shi *et al.*, 2012) was, based on morphological investigations in Razafimandimbison *et al.* (2009*a*), used to constrain the split between Morindeae (here represented by *Morinda citrifolia* L.) and Gaertnereae (here represented by *Gaertnera phyllosepala* Baker). We specified a uniform prior age distribution for the Morindeae stem lineage with a minimum age of 38 Ma (middle–late Eocene; Shi *et al.*, 2012) and a maximum age of 70 Ma. The root node (split between Psychotrieae and Spermacoceae alliances) was constrained to a minimum age of 0 Ma, a mean age of 61 Ma and a standard deviation of 4.6 Ma, which results in 95 % highest probability density (HPD) limits of 52–70 Ma, consistent with results in Wikström *et al.* (2015). A truncated normal prior age distribution was also specified for the Spermacoceae crown group with a minimum age of 0 Ma, a mean age of 48 Ma and a standard deviation of 5.1 Ma, resulting in 95 % HPD limits of 38–58 Ma, consistent with Wikström *et al.* (2015).

The relaxed-clock analyses were otherwise executed in the same way as the non-clock analyses (see above). Consensus trees were produced from the posterior distributions of each of these analyses, as well as from combining them into a single posterior distribution (in order to accommodate the variation in the BPP obtained in the three individual analyses using different clock models) (Supplementary Data Table S3).

## RESULTS

### Main topological results

**Fig. 3.**
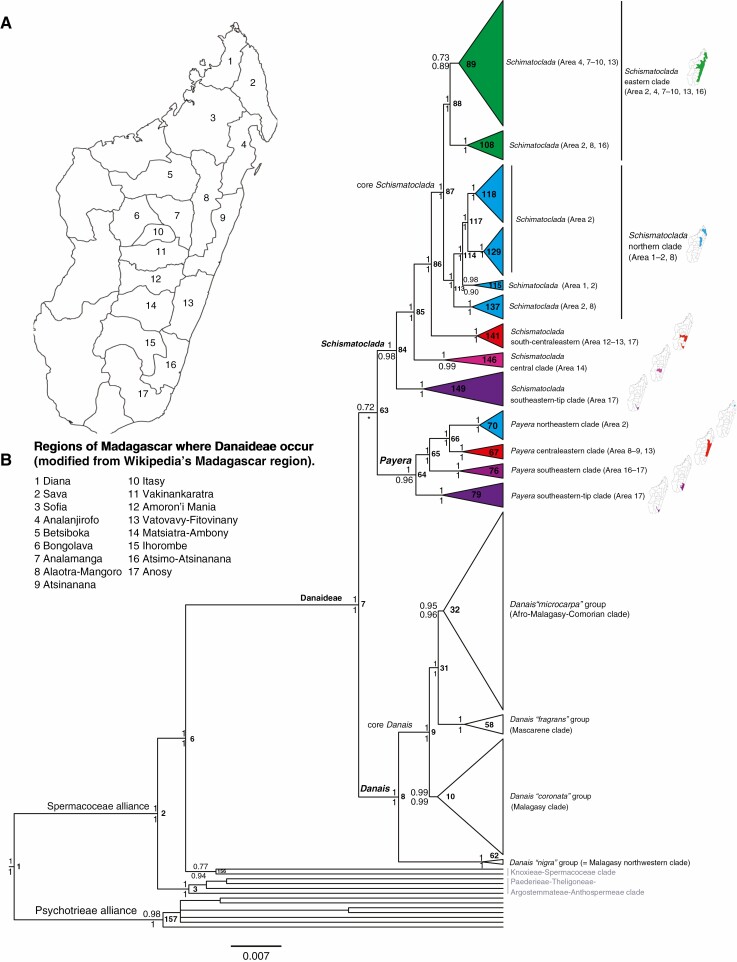
Phylogeny and distribution of Danaideae (Rubiaceae). The regions of Madagascar in which members of Danaideae occur (A). The phylogeny shows a simplified 50% Bayesian majority-rule consensus tree retrieved from the combined tree distributions of three individual relaxed-clock analyses (using three different relaxed-clock models), and based on analyses of plastid *matK*, *ndhF*, *trnT-F* and nuclear ribosomal ITS from 193 samples. Values above and below nodes are Bayesian posterior probabilities from the relaxed-clock and non-clock analyses, respectively. Node numbers are indicated in bold type to the right of nodes. The mini-maps show geographical distributions of clades across the official Regions of Madagascar (B). Scale bar represents expected number of substitutions per site.

**Fig. 4.**
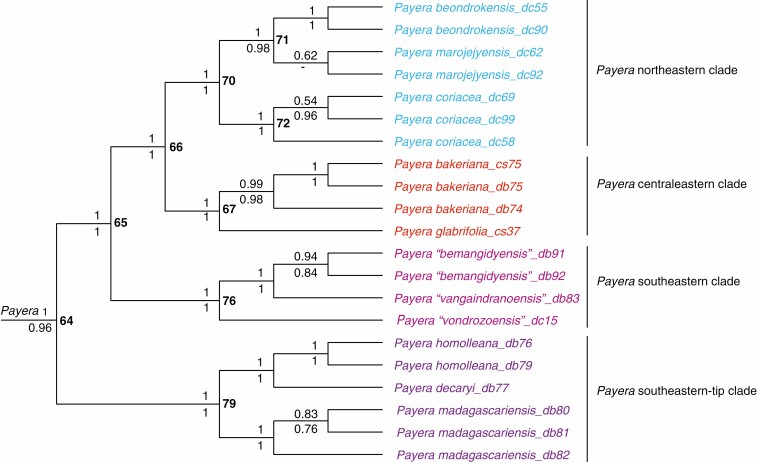
Detailed phylogenetic results from the analysis presented in Fig. 3, drawn as a cladogram, to show relationships in the genus *Payera*. As in Fig. 3, support values above and below nodes are Bayesian posterior probabilities from the relaxed-clock and non-clock analyses, respectively. Node numbers are indicated in bold type to the right of nodes.

**Fig. 5.**
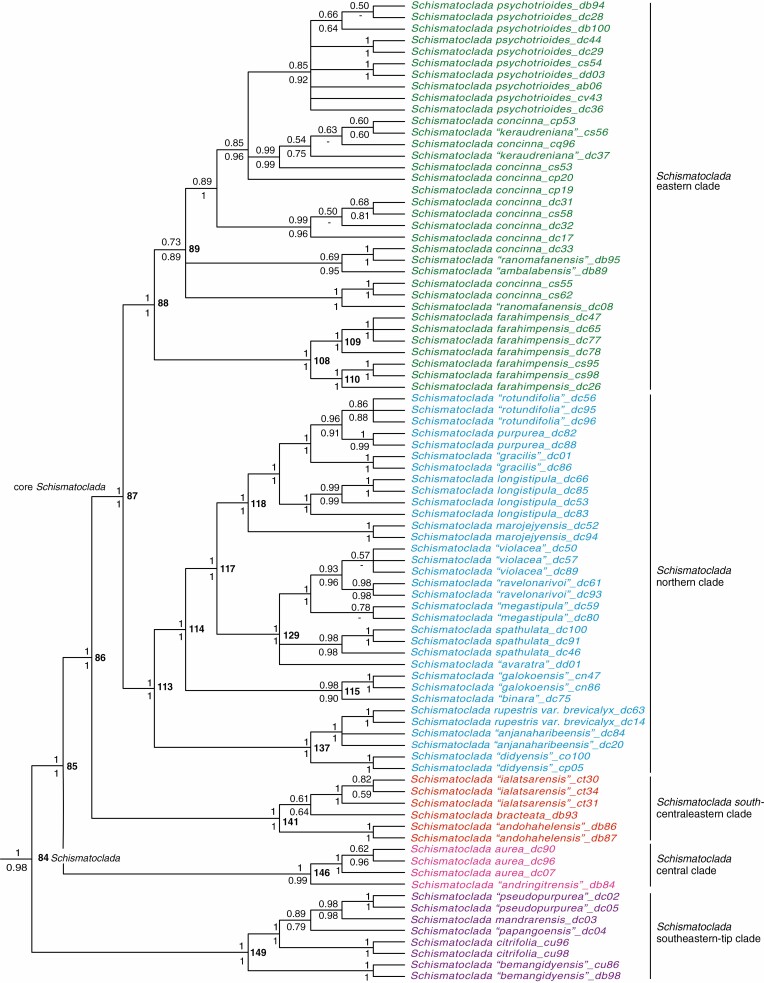
Detailed phylogenetic results from the analysis presented in Fig. 3, drawn as a cladogram, to show relationships in the genus *Schismatoclada*. As in Fig. 3, support values above and below nodes are Bayesian posterior probabilities from the relaxed-clock and non-clock analyses, respectively. Node numbers are indicated in bold type to the right of nodes.

**Fig. 6.**
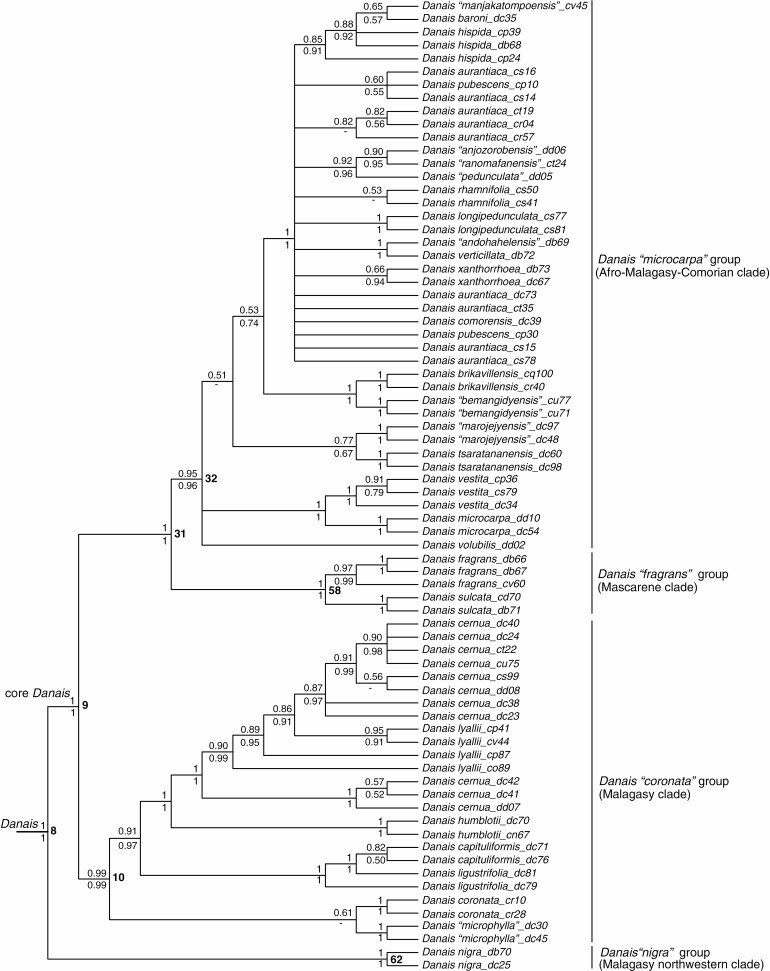
Detailed phylogenetic results from the analysis presented in Fig. 3, drawn as a cladogram, to show relationships in the genus *Danais*. As in Fig. 3, support values above and below nodes are Bayesian posterior probabilities from the relaxed-clock and non-clock analyses, respectively. Node numbers are indicated in bold type to the right of nodes.

Phylogenetic results and clade distributions based on the relaxed-clock analyses are summarized in Fig. 3 with BPP values indicated above nodes and those from the non-clock analyses added below nodes. The Danaideae and each of its three genera are strongly supported as monophyletic. *Danais* is sister to *Payera* + *Schismatoclada* but the statistical support for the latter clade is weak (BPP 0.72). *Danais* is resolved into four main clades, a Malagasy northwestern clade, a clade with broader distribution on Madagascar, a Mascarene clade, and a mostly Malagasy clade with a few species in the Comores and Tanzania (Fig. 3). *Payera* and *Schismatoclada* are resolved into four and five main clades, respectively, which are all geographically restricted to one or a few of the regions of Madagascar (Fig. 3). Detailed phylogenetic results within *Payera*, *Schismatoclada* and *Danais* are, respectively, depicted in Figs 4–6 (see below).

### Morphological studies

We were able to identify all the Danaideae specimens used in our study to species (Supplementary Data Table S2). Our phylogenetic results show that gross morphological features that are frequently discussed in the taxonomic literature on *Payera* and *Schismatoclada* do not characterize monophyletic groups. In *Payera*, stipules can be fimbriate as in *P. homolleana* (Cavaco) R. Buchner & Puff of the *Payera* southeastern-tip clade, *P.* ‘*vangaindranoensis*’ ined. of the *Payera* southeastern clade, and *P. glabrifolia* in the *Payera* centraleastern clade (Fig. 4) (and *P*. ‘*ambohivohitrensis*’ ined.; no molecular data) or entire as in the remaining species of *Payera*. Corolla colour in *Payera* can be yellow as in *P. beondrokensis* and *P. coriacea* of the *Payera* north-eastern clade, purple to dark purple as in *P. marojejyensis* in the *Payera* northeastern clade (and *P.* ‘*ambalabeensis*’ ined.; no molecular data), white corolla tubes with red-orange corolla lobes as in *P.* ‘*vangaindranoensis*’ in the *Payera* southeastern clade (Fig. 4), or white as in the remaining species of *Payera*.

In *Schismatoclada*, leaf size, type and phyllotaxy, stipule type and flower colour are useful for species recognition, but these features do not diagnose monophyletic groups. Leaf size is variable, but species may have relatively small leaves (4–15 mm long × 2–5 mm wide) as in *S.* ‘*papangoensis*’ ined. of the *Schismatoclada* southeastern-tip clade, *S.* ‘*andringitrensis*’ ined. of the *Schismatoclada* central clade, *S.* ‘*didyensis*’ ined., *S.* ‘*binara*’ ined., *S. spathulata*, *S. marojejyensis* and *S.* ‘*gracilis*’ ined. of the *Schismatoclada* northern clade, and *S.* ‘*keraudreniana*’ ined. of the *Schismatoclada* eastern clade (Fig. 5) (and *S.* ‘*ambohivohitrensis*’ ined., *S. humbertiana* Homolle and *S.* ‘*tsaratananensis*’ ined.; no molecular data), or may have larger leaves (at least 25 mm long × more than 5 mm wide) as in the remaining species of *Schismatoclada*. Leaf phyllotaxy is either verticillate as in *S. citrifolia* of the *Schismatoclada* southeastern-tip clade, *S. marojejyensis*, *S.* ‘*megastipula*’ ined., *S.* ‘*ravelonarivoi*’ ined., *S. spathulata* and *S.* ‘*violacea*’ ined. of the *Schismatoclada* northern clade (Fig. 5) (and *S. humbertiana*; no molecular data), or opposite as in the remaining species. Stipules can be either deeply fimbriate as in *S. longistipula*, *S. purpurea* Homolle and *S.* ‘*rotundifolia*’ ined. of the *Schismatoclada* northern clade (Fig. 5) (and *S. aurantiaca*; no molecular data), or entire as in the remaining species of *Schismatoclada*. Corollas are purple or mauve as in *S. citrifolia* of the *Schismatoclada* southeastern-tip clade, and *S.* ‘*megastipula*’, *S.* ‘*ravelonarivoi*’ and *S.* ‘*violacea*’ of the *Schismatoclada* northern clade, yellow to pale yellow as in *S. aurea* Homolle of the *Schismatoclada* central clade, and *S. marojejyensis* of the *Schismatoclada* northern clade, or white as, for example, in *S. ‘binara*’ of the *Schismatoclada* northern clade and *S.* ‘*ialatsarensis*’ ined. of the *Schismatoclada* south-centraleastern clade (Fig. 5), or have white-tinged violate corolla lobes as in the remaining species of the genus.

### Phylogenetic results based on analyses of molecular data

#### Analyses of separate gene regions.

The 50 % Bayesian majority rule consensus trees generated from non-clock analyses of the nrITS, the non-coding plastid (*trnT-F*) and coding plastid (*matK* and *ndhF*) are presented as Supplementary Data (Fig. S1). Visual inspection showed no supported topological conflict (supported clades are defined as having a BPP ≥ 0.95, Erixon *et al.*, 2003). Accordingly, we merged the sequence data of these four markers into a large matrix, which contained a total of 193 samples and 7082 characters, of which 1115 were parsimony-informative. GenBank accessions of the 677 newly produced sequences (ON924473–ON924632, ON937763–ON938105 and ON960373–ON960551) are given in Table S2.

#### Analyses of the combined data set

. Results from the combined non-clock and the three combined relaxed-clock analyses were congruent; none of the observed differences received strong support in either analysis. Figures 3–6 depict the Bayesian 50 % majority rule tree produced from the relaxed-clock analyses of the combined dataset, with the posterior tree distributions of the three relaxed-clock analyses merged into a single distribution. The resulting support values are shown above nodes (Figs 3–6). Support values from the non-clock analysis of the same dataset are plotted below nodes (Figs 3–6). Node numbers and BPP values calculated from the three individual distributions of the relaxed-clock analyses employing different clock models, as well as from the combined tree distribution, are given as Supplementary Data (Table S3).

#### Phylogenetic results.

The tribe Danaideae was monophyletic (BPP retrieved from the combined relaxed-clock/non-clock analyses: 1/1) (node 7 in Fig. 3 and Supplementary Data Table S3), as were the three genera *Payera sensu*Buchner and Puff (1993) (1/0.96) (node 64 in Figs 3–4 and Table S3), *Schismatoclada sensu*Buchner and Puff (1993) (1/0.98) (node 84 in Figs 3 and 5 and Table S3) and *Danais sensu*Puff and Buchner (1994) (1/1) (node 8 in Figs 3 and 6 and Table S3). *Payera* and *Schismatoclada* were poorly supported as sisters in the relaxed-clock analyses (0.72, Fig. 3), whereas *Danais* and *Schismatoclada* were poorly supported as sisters in the non-clock analysis (0.61, data not shown).

*Payera* was almost fully resolved (Fig. 4), and subdivided into four geographically segregated monophyletic groups, all strongly supported (1/1) (Figs 3 and 4). The *Payera* southeastern-tip clade (node 79) and the *Payera* southeastern clade (node 76) were successive sisters to a clade formed by the sister clades the *Payera* centraleastern and northeastern clades (nodes 67 and 70) (Figs 3 and 4). Within the *Payera* southeastern-tip clade (node 79), *P. decaryi* (Homolle) R. Buchner and Puff and *P. homolleana* (1/1) were sisters (1/1) and this latter clade was sister to *P. madagascariensis* (Cavaco) R. Buchner and Puff (1/1) (Fig. 4). Within the *Payera* southeastern clade (node 76), *P.* ‘*bemangidyensis*’ ined. (0.94/0.84) and *P.* ‘*vangaindranoensis*’ were sisters (1/1) and this clade was sister to *P.* ‘*vondrozoensis*’ ined. (1/1). Within the *Payera* central-eastern clade (node 67), *P. glabrifolia* was sister to *P. bakeriana* (Homolle) R. Buchner and Puff (0.99/0.98) (Fig. 4). Within the *Payera* northeastern clade (node 70), *P. marojejyensis* (0.62/-; dash means that the *P. marojejyensis* clade collapses) and *P. beondrokensis* (1/1) formed a clade (1/0.98; node 71), which was sister (1/1; node 70) to *P. coriacea* (1/1; node 72) (Fig. 4 and Supplementary Data Table S3).

*Schismatoclada* was almost fully resolved, and subdivided into five major clades (Figs 3 and 5). The *Schismatoclada* southeastern-tip clade (1/1; node 149), the *Schismatoclada* central clade (1/0.99; node 146) and the *Schismatoclada* south-centraleastern clade (1/1; node 141) were successive sisters to the core *Schismatoclada* (1/1; node 87) formed by the sisters the *Schismatoclada* northern clade (1/1; node 113) and the *Schismatoclada* eastern clade (1/1; node 88) (Figs 3, 5). Within the *Schismatoclada* southeastern-tip clade (node 149), *S.* ‘*bemangidyensis*’ ined. (1/1), *S. citrifolia* (1/1) and S. ‘*papangoensis*’ were successive sisters to a clade (0.98/0.98) formed by the sister species, *S. mandrarensis* and *S.* ‘*pseudopurpurea*’ ined. (1/1). Within the *Schismatoclada* central clade (node 146), *S.* ‘*andringitrensis*’ and *S. aurea* (1/1) were resolved as sisters (1/0.99). Within the *Schismatoclada* south-centraleastern clade (node 141), *S.* ‘*andohahelensis*’ ined. (1/1) was sister to a poorly supported clade (0.61/0.64) formed by *S. bracteata* and *S.* ‘*ialatsarensis*’ (1/1). Within the *Schismatoclada* northern clade (node 113), a northeastern subclade (1/1; node 137) and a northwestern–northeastern subclade (0.98/0.90; node 115) were successive sisters to a strictly northeastern subclade (1/1; node 117) (Figs 3 and 5). Within the northeastern subclade (node 137), *S.* ‘*didyensis*’ (1/1) was sister to a well-supported clade (1/1) formed by *S.* ‘*anjanaharibensis*’ ined. and *S. rupestris* Homolle var. *brevicalyx* Humbert (1/1). Within the *Schismatoclada* northwestern–northeastern subclade (node 115), two species were sisters, *S.* ‘*galokoensis*’ ined. (1/1) and *S.* ‘*binara*’. The northeastern subclade (node 117) is resolved into two sister groups restricted to the Marojejy Massif (with the exception of *S. purpurea* also known from the Tsaratanana Massif): a *Schismatoclada* northeastern group (1/1, node 129) included *S.* ‘*avaratra*’ ined., *S. spathulata* (0.98/0.98), *S.* ‘*megastipula*’ (0.78/-), *S.* ‘*ravelonarivoi*’ (0.98/0.98) and *S.* ‘*violacea*’ (0.57/-). The three latter species formed a monophyletic group (1/1), while the former two species were left unresolved within the clade. A second northeastern group (1/1; node 118) contained *S. marojejyensis* (1/1), *S. longistipula* (1/1) and *S.* ‘*gracilis*’ (1/1), which were successive sisters to a clade (0.96/0.91) comprising *S. purpurea* (1/0.99) and *S.* ‘*rotundifolia*’ (0.86/0.88). Within the *Schismatoclada* eastern clade (1/1; node 88), *S. farahimpensis* (1/1; node 108) was sister to a poorly supported clade with a relatively broad distribution in eastern Madagascar (0.73/0.89; node 89), which contained the type of *Schismatoclada*, *S. psychotrioides* (BPP 0.85/0.92), and (the apparently non-monophyletic) *S. concinna*, as well as *S.* ‘*ambalabeensis*’ ined., *S.* ‘*keraudreniana*’ and *S.* ‘*ranomafanensis*’ ined. Relationships among the five taxa of this clade (node 89) were generally poorly supported. Relationships among samples of *S. farahimpensis* (node 108) were, however, well resolved (Figs 3 and 5 and Supplementary Data Table S3).

*Danais* was resolved into four well-supported major clades (Figs 3 and 6). The *Danais* ‘*nigra*’ group (1/1; node 62) from northwestern Madagascar was sister to the core *Danais* (1/1; node 9), which comprised the *Danais* ‘*coronata*’ group *sensu*Puff and Buchner (1994), i.e. the Malagasy clade (0.99/0.99; node 10), sister to a clade (1/1; node 31) comprising the *Danais* ‘*fragrans*’ group, i.e. the Mascarene clade (1/1; node 58) and the *Danais* ‘*microcarpa*’ group *sensu*Puff and Buchner (1994), i.e. the Afro-Malagasy-Comorian clade (0.95/0.96; node 32) (Figs 3 and 6 and Supplementary Data Table S3). Within the *Danais* ‘*coronata*’ group (node 10), *D. coronata* (Pers.) Steud. (1/1) + *D.* ‘*microphylla*’ ined. (1/1), a *D. capituliformis*–*D. ligustrifolia* clade (1/1) and *D. humblotii* Homolle (1/1) were successive sisters to a *Danais cernua*–*D. lyallii* clade (1/1). Within the Mascarene *Danais* ‘*fragrans*’ group (node 58), the Mauritian–Reunionese species *D. fragrans* (0.97/0.99) and *D. sulcata* (1/1) were sisters. Phylogenetic relationships within the Afro-Malagasy-Comorian *D.* ‘*microcarpa*’ group (node 32) were largely unresolved, but the following four clades were retained: *D. microcarpa* Baker (1/1) and *D. vestita* Baker (1/1) were sisters (1/1); *D. tsaratananensis* Homolle (1/1) and *D.* ‘*marojejyensis*’ ined. (1/1) were sisters (0.77/0.67); *D.* ‘*bemangidyensis’* ined. (1/1) and *D. brickavillensis* J.-F. Leroy ex Puff & R. Buchner (1/1) were sisters (1/1) and sister to a large clade (1/1) formed by 28 samples from 12 Malagasy *Danais* species, as well as the Comorian *D. comorensis* Drake (Drake, 1889) and the Tanzanian *D. xanthorrhoea* (0.66/0.94) (Figs 3 and 6 and Table S3).

### Species monophyly

A total of 42 species and one variety of Danaideae were resolved as monophyletic: seven species of *Payera* (*P. madagascariensis*, *P. homolleana*, *P.* ‘*bemangidyensis*’, *P. bakeriana*, *P. coriacea*, *P. marojejyensis*, *P. beondrokensis*) (Fig. 4); 19 species and one variety of *Schismatoclada* [*S.* ‘*bemangidyensis*’, *S. citrifolia*, *S.* ‘*pseudopurpurea*’, *S. aurea*, *S.* ‘*andohahelensis*’, *S.* ‘*ialatsarensis*’, *S.* ‘*didyensis*’, *S.* ‘*galokoensis*’, *S. spathulata*, *S.* ‘*megastipula*’, *S.* ‘*ravelonarivoi*’, *S.* ‘*violacea*’, *S. marojejyensis*, *S. longistipula*, *S.* ‘*gracilis*’, *S. purpurea*, *S.* ‘*rotundifolia*’, *S. farahimpensis*, *S. psychotrioides* and *S. rupestris* var. *brevicalyx* (= *S. rubra* Homolle var. *brevicalyx* (Humbert) Cavaco)] (Fig. 5); and 16 species of *Danais* (*D. nigra* Homolle, *D.* ‘*microphylla*’, *D. coronata*, *D. capituliformis* Homolle, *D. humblotii*, *D. sulcata*, *D. fragrans*, *D. microcarpa*, *D. vestita*, *D. tsaratananensis*, *D.* ‘*marojejyensis*’, *D.* ‘*bemangidyensis*’, *D. brickavillensis*, *D. xanthorrhoea*, *D. longipedunculata* Homolle and *D. rhamnifolia* Baker) (Fig. 6).

Three species of *Schismatoclada* (*S. concinna*, *S.* ‘*keraudreniana*’ and *S.* ‘*ranomafanensis*’) are non-monophyletic as assessed from our data (Fig. 5). Six species of *Danais* were also resolved as non-monophyletic (*D. ligustrifolia* Baker, *D. cernua*, *D. lyallii*, *D. aurantiaca*, *D. pubescens* Baker and *D. hispida* Baker; Fig. 6).

## DISCUSSION

### Generic monophyly in Danaideae

The monophyly of the tribe Danaideae *sensu*Bremer and Manen (2000) is supported by all separate and combined analyses; our combined nuclear–plastid analyses (Figs 3–6) are the first to demonstrate with strong support (1/1) the monophyly of all the three Danaideae genera, *Danais sensu*Puff and Buchner (1994), and *Payera* and *Schismatoclada* both *sensu*Buchner and Puff (1993). Buchner and Puff (1993) formally transferred four arborescent Malagasy species of *Danais* with pubescent corollas (*D. bakeriana*, *D. decaryi*, *D. madagascariensis* and *D. mandrarensis*, all *sensu*Cavaco, 1966) to *Payera* [*P. bakeriana*, *P. decaryi*, *P. madagascariensis* and *P. mandrarensis* (Homolle ex Cavaco) R. Buchner & Puff], and this taxonomic decision is strongly supported by our results (Fig. 4). *Danais mandrarensis* was not investigated in the present study but we expect it to belong to *Payera*, as it has some of the salient features of the genus (e.g. valvate–reduplicate corolla aestivation and pubescent corollas). The mutual paraphyly of *Payera* and *Schismatoclada* suggested by Krüger *et al.* (2012) is mainly explained by misidentifications of many specimens of *Payera* and *Schismatoclada* in that study.

The inclusion of the Malagasy monotypic genus *Coursiana*, represented by *C. homolleana*, in *Payera* (i.e. *Payera homolleana*) proposed by Buchner and Puff (1993) receives strong support from the results of the present study (Fig. 4). On the other hand, *Schismatoclada* as delimited by Humbert (1955) and Cavaco (1964), both of which included the two low-growing shrubby species *Schismatoclada beondrokensis* (now *Payera beondrokensis*) and *Schismatoclada coriacea* (now *Payera coriacea*), is not supported by our analyses. These latter species are shown here to belong to *Payera*, consistent with Buchner and Puff (1993) and Krüger *et al.* (2012), and are resolved in the *Payera* northeastern clade (Fig. 4).

### Phylogenetic relationships among the Danaideae genera

While all three genera of Danaideae are, for the first time, strongly supported as monophyletic in our study, their interrelationships remain unresolved. The sister-relationship between *Payera* and *Schismatoclada* received poor support (BPP 0.72) in the relaxed-clock analysis (Fig. 3), and the non-clock analysis instead provided weak support (BPP 0.61) for a sister-relationship between *Danais* and *Schismatoclada*. The difficulty in resolving the generic relationships in Danaideae with molecular data goes in concert with the conflicting generic relationships indicated by morphology (e.g. Buchner and Puff, 1993; Schatz, 2001). For example, the arborescent habit of *Payera* and *Schismatoclada* (as opposed to lianescent habit in *Danais*) seems to suggest their close affinity. By contrast, the capsular fruits of *Danais* and *Payera* have loculicidal dehiscence, whereas those of *Schismatoclada* bear septicidal dehiscence (Buchner and Puff, 1993). Furthermore, *Danais* and *Schismatoclada* appear more closely related to each other than either is to *Payera* based on the absence of the large bracts subtending the inflorescence (vs. mostly present in *Payera*), valvate–reduplicate corolla aestivation (vs. valvate in *Payera*) and glabrous corolla (vs. pubescent or puberulous in *Payera*).

#### Phylogenetic relationships in Payera.

Our analyses resolved *Payera* in four well-supported monophyletic groups (Figs 3B and 4). The first lineage to diverge, the *Payera* southeastern-tip clade, contains at least three morphologically distinct species of shrubs (*P. decaryi*, *P. homolleana* and *P. madagascariensis*) (Fig. 4) that grow sympatrically in the lowland rainforests of the Anosy Region (Fig. 3A: Area 17). *Payera decaryi* can easily be distinguished from the other two by its very large stipules and leaves, long-pedunculate, pendulous, racemose inflorescences, and flowers with white corolla tubes but orange corolla lobes. Both *P. homolleana* and *P. madagascariensis* have globose inflorescences but the former has relatively short peduncles, while the latter has pendulous, long peduncles up to 10 cm long. *Payera decaryi* and *P. madagascariensis* are endemic to this region, while *P. homolleana sensu*Buchner and Puff (1993) is known from the Anosy Region and the Alaotra-Mangoro Region (Fig. 3A: Area 8). While the samples of *P. homolleana* included in this study are from the Anosy Region, the type specimens of the species are from the Ambatondrazaka District in the Alaotra-Mangoro Region. Buchner and Puff (1993) treated the populations of *P. homolleana* from the Anosy and Alaotra-Mangoro Regions as the same species mainly because of their densely pubescent habits, but we do not necessarily expect them to form a monophyletic group. They can easily be distinguished based on their inflorescence types and the colour of their corollas (purple vs. white), and that they are geographically distinct. If future work based on molecular data confirms the populations of the Anosy and Alaotra-Mangoro Regions as distantly related, *P. homolleana* from the southeast tip would have to be described as a new species.

The next lineage to diverge, the *Payera* southeastern clade (Figs 3B and 4), encompasses at least three morphologically distinct species of shrubs (*P.* ‘*vondrozoensis*’ sister to *P.* ‘*vangaindranoensis*’ and *P.* ‘*bemangidyensis*’) (Fig. 4). The three species are newly proposed, have yet to be described and appear not to be sympatric. *Payera bemangidyensis* grows together with *P. homolleana* and *P. madagascariensis* in the Anosy Region (Fig. 3A: Area 17), whereas *P.* ‘*vangaindranoensis*’ and *P.* ‘*vondrozoensis*’ are known only from separate districts within the Atsimo Atsinanana Region (Fig. 3A: Area 16) in southeastern Madagascar. We suspect that a fourth undescribed species (*P.* ‘*ambodivohitrensis*’ ined.), only known from the summit of the Ambodivohitra mountain (1200–1400 m) of the Vohibato District within the Matsiatra–Ambony Region (Fig. 3A: Area 14) belongs in the *Payera* southeastern clade. This new species appears to be closely related to *P.* ‘*bemangidyensis*’ and *P.* ‘*vangaindranoensis*’ based on its narrowly lanceolate, membranous leaves, but differs from the other two by having much smaller leaves, fimbriate stipules and subsessile head-like inflorescences bearing orange corollas. *Payera* ‘*bemangidyensis*’ has bifid stipules and rather lax inflorescences/infructescences with pendulous fruits, and its leaf size is intermediate between that of *P.* ‘*ambohivohitrensis*’ and *P.* ‘*vangaindranoensis*’. *Payera* ‘*vangaindranoensis*’ is known from the Manombo Reserve (Farafangana District) and the Vohipaho forest (Vaingaindrano District) within the Atsimo-Atsinanana Region (Fig. 3A: Area 16). This species differs from the other species in the *Payera* southeastern clade by its deeply fimbriate stipules and globose inflorescences bearing orange corollas. *Payera* ‘*vondrozoensis*’ is known from a single collection (*De Block et al. 1979*, BR) from the Vondrozo District in the Atsimo Atsinanana Region (Fig. 3A: Area 16). It is morphologically similar to *P. homolleana* but differs by its large stipules with laciniate margins and subsessile flowers and fruits (as opposed to stipules with laciniate margins and long-pedicellate flowers and fruits in *P. homolleana*).

The *Payera* centraleastern clade (Figs 3B and 4) contains at least two morphologically distinct species, *P. bakeriana* and *P. glabrifolia*, with adjacent geographical ranges. These two sister species can easily be distinguished morphologically; *P. bakeriana* has narrowly lanceolate leaves, small stipules and lax inflorescences, while *P. glabrifolia* has broadly lanceolate, glabrous leaves that are large with laciniate margins, and condensed inflorescences. *Payera bakeriana* has a wide distribution in the highland rainforests, ranging from the Andringitra Massif (Matsiatra-Ambony Region, Fig. 3A: Area 14), through the Ranomafana National Park (Ifanadiana District, Vatovavy Fitovinany Region, Fig. 3A: Area 13), to the Moramanga and Ambatondrazaka Districts (Mangoro-Alaotra Region, Fig. 3A: Area 8). Many remote areas of Madagascar have not yet been thoroughly searched by botanists, and we expect the species to also occur in the Marolambo and Anosibe An’ala Districts (Atsinanana Region, Fig. 3A: Area 9), which lie between Ifanadiana and the Moramanga Districts in the central east of Madagascar. *Payera glabrifolia* is known from the lowland rainforests in the Brickaville, Toamasina II and Vatomandry Districts (Atsinanana Region, Area 9), through the Ambatondrazaka District (Alaotra-Mangoro Region, Fig. 3A: Area 8) to the Maroantsetra District, and we expect it to occur also in the Soanerana-Ivongo and Mananara-Avaratra Districts (Analanjirofo Region, Fig. 3A: Area 4) although it has not yet been discovered from that area.

The *Payera* northeastern clade (Figs 3B and 4) encompasses at least three species of ericoid shrubs (*P. coriacea*, sister to *P. beondrokensis*, and *P. marojejyensis*) (Fig. 4). These species grow exclusively and sympatrically in the ericoid thickets near the summit of the Marojejy Massif in northeastern Madagascar (Figs 1 and 3A: Area 2), and are morphologically distinct. While they are all low-growing shrubs up to 50 cm tall, *P. marojejyensis* has dark purple flowers as opposed to yellow in the other two species. *Payera coriacea* has large, persistent, appressed stipules and glabrous leaves, whereas *P. beondrokensis* bears small stipules and pubescent leaves.

#### Phylogenetic relationships in Schismatoclada.

Our analyses resolve *Schismatoclada* in five major lineages (Figs 3B and 5). The *Schismatoclada* southeastern-tip clade, sister to the remainder of the genus, comprises at least five species (*S.* ‘*bemangidyensis*’, *S. citrifolia*, *S.* ‘*papangoensis*’, *S. mandrarensis* and *S.* ‘*pseudopurpurea*’, Fig. 5), which are restricted to the Fort-Dauphin District within the Anosy Region (Fig. 3A: Area 17). Based on their geographical proximity to these five species, we postulate that *S. rupestris* var. *rupestris* and *S. rubra* belong to this lineage as well (see below). Cavaco (1967) merged *S. rubra* with *S. citrifolia* and Buchner and Puff (1993) endorsed this taxonomic adjustment, but we disagree with this assertion based on both morphology and geography (see below), although molecular data from *S. rubra* are currently unavailable to us to confirm this. *Schismatoclada citrifolia* is confined to the sandy littoral forests, and it also frequently grows along or in the swampy areas north of Fort Dauphin town (e.g. Ampangalatsilo, Etazo, Mandena, Saint Luce). *Schismatoclada citrifolia* is similar to *S. rupestris* (i.e. *S. rupestris* var. *rupestris*) in its verticillate leaves, cymose inflorescences and mauve corollas. However, the latter is distinct by having smaller, coriaceous leaves with revolute margins, connate stipules with triangular appendages and foliaceous but shorter calyx lobes (as opposed to larger non-coriaceous leaves with non-revolute margins, triangular stipules and foliaceous, longer calyx lobes in the latter). Furthermore, *S. rupestris* is a mountain species (1300–1850 m in altitude) known from the Ivakoany and Kalambatitra Massifs of the Anosy mountain chains, and its geographical ranges are quite distant from that of *S. citrifolia*. We were unfortunately unable to sample representatives of *S. rupestris* var. *rupestris*; however, based on the evidence shown in this study, we expect it to belong to the *Schismatoclada* southeastern-tip clade (and thus not to group with *S. rupestris* var. *brevicalyx* resolved in the *Schismatoclada* northern clade, see further below)*. Schismatoclada* ‘*bemangidyensis*’, strongly supported as sister to the remaining four species in the clade, is a new undescribed species known only from two collections in fruit from the lowland rainforest of Bemangidy in the northern part of the Tsitongabarika new protected area. It can be distinguished by its glossy, broad, glabrous obovate leaves and glossy young, beaked, elongated fruits ornamented by small triangle calyx lobes.

*Schismatoclada* ‘*papangoensis*’ is restricted to the ericoid thicket at the summit of the Papanga Mont, one of the satellites of the Beampingaratra Massif of the Anosy mountain chains. It is distinct from the other members of the southeastern-tip clade by its relatively small elliptical leaves. This species, yet to be described, is only known from a single specimen collected by Humbert in 1928, and has not been recollected since. *Schismatoclada mandrarensis* was collected from the same locality, but the species has a wider range and is known from more collections than *S.* ‘*papangoensis*’. It is worth noting that Cavaco (1964) considered *S. mandrarensis* and *S. aurantiaca* to be conspecifics, and *S. mandrarensis* is currently not an accepted species name. *Schismatoclada aurantiaca* is, as defined by Homolle (1939), restricted to the Andringitra Massif (Matsiatra-Ambony Region, Fig. 3A: Area 14) and the Ivohibe Massif (Atsimo-Atsinanana Region, Fig. 3A: Area 16). It differs from *S. mandrarensis* by its membranaceous leaves and relatively large paniculate inflorescences (Homolle, 1939), as opposed to coriaceous leaves and smaller paniculate inflorescences in *S. mandrarensis*, and we have therefore (provisionally) treated the two as distinct species. We expect *S. aurantiaca* to belong in the *Schismatoclada* central clade given that its geographical range overlaps with that of *S. aurea*. However, no sample of *S. aurantiaca* from its type locality (Andringitra Massif) was analysed in this study, and whether these two species are conspecifics remains to be tested with molecular data. Finally, *S.* ‘*pseudopurpurea*’ is known only from two specimens from the summit of the Ambatofotsy Mont within the Andohahela Massif in the Anosy Region (Fig. 3A: Area 17). The leaf shape and venation of this species are similar to those of *S. purpurea*, but *S. purpurea* is restricted to the Tsaratanana and Marojejy Massifs (Fig. 3A: Areas 1 and 2) in northern Madagascar and belongs in the *Schismatoclada* northeastern clade (Figs 3B and 5).

The *Schismatoclada* central clade (Figs 3B and 5) is the second clade to diverge, and it encompasses at least two species (*S.* ‘*andringitrensis*’ and *S. aurea*) (Fig. 5). The former species is known from two specimens from the highland rainforests of both the Andringitra and Ambondrombe Massifs (Ambalavao District) within the Matsiatra-Ambony Region (Fig. 3A: Area 14). By contrast, the latter species is restricted to high elevations of the Andringitra Massif and the Pic Ivohibe National Park (Vondrozo District, Atsimo Atsinanana Region, Fig. 3A: Area 16). *Schismatoclada aurea* has red stems, yellow–orange corollas (or whitish tubes and orange lobes) and globose fruits, as has *S. homolleae.* Based on morphology, we argue that *S. homolleae* and *S. aurea* are conspecifics. *Schismatoclada* ‘*andringitrensis*’ is markedly distinct among species of the central clade by its winged stems, small, spathulate leaves, and its long-pedunculate, elongate fruits with longitudinal ridges. Its flowers are unknown. *Schismatoclada pubescens* Cavaco is distinct by its pubescent stems, leaves and corollas. It was not sampled for the present study; it is confined to the lowland swampy forests in the Vondrozo District (Atsimo-Atsinanana Region, Fig. 3A: Area 16), and is known only from three specimens. However, based on the general phylogenetic patterns of *Schismatoclada* as revealed by our results, we postulate that *S. pubescens* belongs in the *Schismatoclada* central clade.

The *Schismatoclada* south-centraleastern clade (Figs 3B and 5), the next clade to diverge, contains at least three species (*S. bracteata* and the new undescribed species *S.* ‘*andohahelensis*’ and *S.* ‘*ialatsarensis*’; Fig. 5). *Schismatoclada bracteata* is known to occur in the Andringitra Massif (District Ambalavao, Matsiatra-Ambony Region, Fig. 3A: Area 14), the Ranomafana National Park (Ifanadiana District, Vatovinany-Fitovavy Region, Fig. 3A: Area 13), whereas *S.* ‘*andohahelensis*’ is known only from two recent collections from the north of the Tsitongabarika new protected area (Anosy Region, Fig. 3A: Area 17). Similarly, *S. ialatsarensis* is only known from two recent collections from the Ialatsara forest of the Ambohimahasoa District (Matsiatra-Ambony Region). The placement of *S.* ‘*andohahelensis*’ in this clade is strongly supported by molecular data but deviates from the geographical patterns of the major lineages of *Schismatoclada*, as shown in this study.

The *Schismatoclada* northern clade of the core *Schismatoclada* (Figs 3B and 5) is a species-rich clade formed by three geographically segregated subclades: clade 137 distributed in Areas 2 and 8; clade 115 distributed in Areas 1 and 2; and clade 117 distributed in Area 2 (Fig. 3A). Clades 137 and 115 are successive sisters to clade 117, which in turn is divided into clades 129 and 118, both distributed in Area 2 (Fig. 3A). The *Schismatoclada* clade 137 contains at least three species (*S.* ‘*didyensis*’ sister to *S*. ‘*anjanaharibeensis*’ ined. + *S. rupestris* var. *brevicalyx*) (Fig. 5). Based on morphology, we further expect *S. lutea* and the undescribed new species *S.* ‘*makira*’ ined. from the eastern Sofia Region (Fig. 3A: Area 3) and the Analanjirofo Region (Fig. 3A: Area 4) to belong to this subclade. The clade (137) has a disjunct distribution; *S.* ‘*anjanaharibeensis*’ is from the Anjanaharibe-Sud and Marojejy Massifs in the Sava Region (Fig. 3A: Area 2) in northeastern Madagascar, whereas *S.* ‘*didyensis*’, distinguished by its relatively small leaves, sessile head-like inflorescences and white slightly zygomorphic corolla tubes, is known only from the Didy forest of the Ambatondrazaka District within the Alaotra-Mangoro Region (Fig. 3A: Area 8). All other species of clade 137 have much larger leaves, and loose, sessile inflorescences. *Schismatoclada rupestris* var. *brevicalyx* was described by Cavaco (1967) (transferred at the varietal level from *S. rubra* var. *brevicalyx* Humbert), as an endemic to the Marojejy Massif in northeastern Madagascar (Sava Region, Fig. 3A: Area 2). The decision was seemingly justified based on morphological resemblance with *S. rupestris* (e.g. broadly elliptical leaves, short calyx lobes) even though it made *S. rupestris* the sole species of *Schismatoclada* with a disjunct distribution (since the type specimens of *S. rupestris* var. *rupestris* are all from the Beampiangatra Massif). However, while *S. rupestris* var. *brevicalyx* is resolved in the *Schismatoclada* northern clade (clade 137), based on geographical proximity we anticipate that *S. rupestris* var. *rupestris* belongs in the *Schismatoclada* southeastern-tip clade, and we consequently argue that *S. rupestris* var. *brevicalyx* should be recognized at the species level: *S.* ‘*brevicalyx*’ ined.

The *Schismatoclada* northern clade 115 contains at least two undescribed species: *S*. ‘*binara*’, from the Binara forest within the Daraina Commune of the Vohemar District (Sava Region, Fig. 3A: Area 2), and *S.* ‘*galokoensis*’, from the Galoko Massif in the Ambilobe District (Diana Region, Fig. 3A: Area 1). The geographical ranges of these species correspond to the northern and northwestern limits of *Schismatoclada*. It is important to note that the geographical range of *S.* ‘*binara*’ in clade 115 is quite far from that of the species endemic to the Marojejy Massif in the northeastern clade 117, which occur within the Andapa District (Sava Region, Fig. 3A: Area 2). *Schismatoclada* ‘*binara*’ and *S.* ‘*galokoensis*’, both in clade 115, are distinct based on their leaf size and shape. *Schismatoclada* ‘*binara*’ has relatively small, spathulate leaves and long, white corollas, while *S. galokoensis* has medium-sized, elliptical leaves and short white corollas. We expect *S*. ‘*manongarivoensis*’ ined. from the Manongarivo Massif (District Ambanja, Diana Region, Fig. 3A: Area 1) in northwestern Madagascar to be closely related to *S*. ‘*galokoensis*’ given their geographical proximity. The members of the *Schismatoclada* northeastern clade 117 (comprising the sister clades 129 and 118) grow sympatrically between 1000 and 2000 m altitudes in the Beondroka Mont of the Marojejy Massif, with the exception of *S. marojejyensis* which is restricted to the ericoid thicket near the summit of the Marojejy Massif (2136 m in altitude). The *Schismatoclada* clade 129 contains at least five morphologically distinct taxa, *S.* ‘*avaratra*’, *S. spathulata*, *S.* ‘*megastipula*’, *S.* ‘*ravelonarivoi*’ and S. ‘*violacea*’ (Fig. 5). The three latter species form a well-supported monophyletic group that is distinct based on their head-like inflorescences. The *Schismatoclada* northeastern clade 118 includes five morphologically distinct species, *S. marojejyensis*, sister to *S. longistipula*, *S.* ‘*gracilis*’, *S. purpurea* and *S.* ‘*rotundifolia*’ (Fig. 5). Both *S. marojejyensis* and *S.* ‘*gracilis’* have small leaves, but the former differs by being a low-growing shrub, its verticillate, coriaceous, subsessile leaves, persistent stipules, and very long, solitary and pendulous, light-yellow flowers, as opposed to opposite, membranaceous, petiolate leaves and cream flowers arranged in lax, pedunculate inflorescences. *Schismatoclada longistipula* is distinct based on its large leaves and fimbriate, persistent stipules, large paniculate inflorescences and small obovoid fruits. *Schismatoclada purpurea* and *S.* ‘*rotundifolia*’ have ovoid and coriaceous leaves.

The fifth clade, the *Schismatoclada* eastern clade of the core *Schismatoclada* (Figs 3B and 5), includes samples from six currently accepted and newly proposed species: *S. farahimpensis*, *S.* ‘*ranomafanensis*’, *S. concinna*, *S.* ‘*ambalabeensis*’, *S.* ‘*keraudreniana’* and *S. psychotrioides* (Fig. 5). All samples of *S. farahimpensis* form a monophyletic group (clade 108, Fig. 5) even though this species is widely distributed. It is the only widespread species of *Schismatoclada* with a geographical distribution ranging from the southeast tip (Beampiangatra Massifs, Anosy Region, Fig. 3A: Area 17) to northeastern (Marojejy and Anjanaharibe Sud Massifs, Sava Region, Fig. 3A: Area 2) and northwestern regions (Manongarivo Massif, Diana Region, Fig. 3A: Area 1). It is recognized by its membranaceous leaves and persistent triangular stipules covered by glands on its margins and yellow–orange corollas, and our results support two geographically distinct groups of *S. farahimpensis*, the northeastern group (Fig. 3A: Area 2) and the south-central eastern group (Fig. 3A: Areas 8 and 16). *Schismatoclada psychotrioides* is recorded from the Andramasina, Anjozorobe and Ankazobe Districts (Analamanga Region, Fig. 3A: Area 7), the Moramanga District (southern Alaotra-Mangoro, Fig. 3A: Area 8), the Marolambo District (northern Atsinanana Region, Fig. 3A: Area 9) and Ambatolampy District (northern Vakinankaratra Region, Fig. 3A: Area 11). This species is recognized by a combination of the following characters: elliptical leaves with reticulate venation, small triangular, paniculate inflorescences with each axis subtended by a pair of leaf-like bracts, flowers with enlarged cream corolla tubes that are tinged violet outside of the corolla lobes, lanceolate calyx lobes, fruits beaked and capsular, and seeds broadly winged at both ends but narrowly winged along both lateral sides. The monophyly of each of the remaining species of the *Schismatoclada* eastern clade (*S.* ‘*ranomafanensis*’, *S. concinna*, *S.* ‘*ambalabeensis*’, *S.* ‘*keraudreniana*’) is not supported in our results and their respective delimitations need further research.

*Phylogenetic relationships in* Danais. *Danais sensu*Puff and Buchner (1994) is resolved into four well-supported lineages (Figs 3B and 6): the northwestern Malagasy *Danais* ‘*nigra*’ group; the Malagasy *Danais* ‘*coronata*’ group; the Mascarene *Danais* ‘*fragrans*’ group; and the Afro-Malagasy-Comorian *Danais* ‘*microcarpa*’ group, which is centred on Madagascar but expands its geographical ranges to Tanzania (*D. xanthorrhoea*) and the Comores (*D. comorensis*). Also, the *Danais* ‘*coronata*’ group has a wide geographical range with many of its species widely distributed.

*Danais nigra* as defined in our study is resolved as sister to a very large clade formed by the remaining sampled *Danais* (core *Danais*, Figs 3B and 6). This is consistent with Krüger *et al.* (2012), although specimen *Danais nigra* with labID dc25 (voucher *Kårehed et al. 254*) included in both studies was not identified at the species level in Krüger *et al.* (2012) but was referred to as *Danais* sp. 1 in their work. According to Puff and Buchner (1994), *Danais nigra* has a disjunct distribution, the Sambirano area (including Manongarivo and Galoko-Kalobenono Massifs) within the Diana Region (Fig. 3A: Area 1) in northwestern Madagascar and the Masoala National Park and the neighbouring forests, all within Analanjirofo Region (Fig. 3A: Area 4) in the central-northeast of the island. We disagree with this assertion and argue that *D. nigra* is restricted and endemic to the lowlands between the Ambanja and Ambilobe Districts and around the Galoko-Kalabenono Massif within the Diana Region (Fig. 3A: Area 1), because the type specimens (lectotype and isolectotypes) are from there. *Danais nigra* as delimited here is distinct from the so-called *D. nigra* plants from the central-northeastern region (Fig. 3A: Area 4) by having very lax inflorescences and flowers with very long pedicels and erect corolla lobes as opposed to much denser inflorescences and flowers with much shorter pedicels and recurved corolla lobes in the latter plants.

The *Danais* Mascarene clade was poorly supported (BPP 0.84) as sister to the *Danais* ‘*coronata*’ group in Krüger *et al.* (2012), while it is strongly supported as sister to the *Danais* ‘*microcarpa*’ group in the present study. The *Danais* ‘*microcarpa*’ group as defined by Puff and Buchner (1994) includes *D. breviflora* Baker, *D. ligustrifolia*, *D. microcarpa*, *D. rhamnifolia* and *D. verticillata* Baker, and is characterized by their terminal inflorescences, small flowers, fruits and seeds. This group is not monophyletic in our results; many species with large flowers, fruits and seeds are nested in this lineage and *D. ligustrifolia* is instead included in the *Danais* ‘*coronata*’ group (Fig. 6). Buchner and Puff (1993) also postulated *D. brickavillensis*, *D. humblotii*, *D. longipedunculata* and *D. rubra* to be closely related, but this is not supported by our results, as *D. brickavillensis* and *D. longipedunculata* fall in the *Danais* ‘*microcarpa*’ group whereas *D. humblotii* is included in the *D.* ‘*coronata*’ group (Fig. 6). Furthermore, Buchner and Puff (1993: 53) postulated close affinities between *D. coronata*, *D. sulcata* and *D. volubilis* Baker on the basis of their axillary inflorescences, much elongated calyx lobes and rather large fruits. We find no support for this group either, because *D. sulcata* and *D. volubilis* are nested in the *Danais* ‘*fragrans*’ and *Danais* ‘*microcarpa*’ groups, respectively (Fig. 6).

### Species delimitation in Danaideae genera

Taxonomists identify morphologically diagnosable units using presumably autapomorphic characters. From a phylogenetic standpoint, these morphologically diagnosable units (also known as morphospecies) are viewed as species hypotheses, which can be tested using analyses of other sources of data (e.g. molecular data). The result may reveal members of these hypothesized morphospecies as either monophyletic or non-monophyletic units (Rosell *et al.*, 2010). When the result is the latter, non-monophyletic units, it is likely that the presumably autapomorphic morphological characters that were considered diagnostic of a species in reality comprised a combination of autapomorphic, synapomorphic and pleisiomorphic characters (e.g. Razafimandimbison, 2002), meaning that the included specimens represent an assemblage of non-related individuals that would be better understood as several different species. Our taxon sampling allows us to test the monophyly of many species of *Danais*, *Payera* and *Schismatoclada*, both currently accepted and new species proposed here.

We find that 42 species of Danaideae (including one currently described as a variety) are supported as monophyletic (see specification in the result section): seven species of *Payera*, 19 species and one variety of *Schismatoclada*, and 16 species of *Danais* (Figs 4–6). Of these 42, 15 species are yet to be formally described. By contrast, *S. concinna* and *S.* ‘*ranomafanensis*’ are polyphyletic, and possibly *S.* ‘*keraudreniana*’ too although the results are poorly supported. The morphologically variable *S. concinna* as defined by Baker (1885) may well be considered a ‘morphospecies’, members of which do not descend from a common ancestor. The non-monophyly of *S.* ‘*keraudreniana*’ is surprising given that this species is notably distinct from the other species in the same clade by its quadrangular, reddish stems, small spathulate leaves with inconspicuous secondary and tertiary veins, and reduced and sessile inflorescences with one to two flowers with white-greenish corolla tubes and white corolla lobes. Regarding *Danais*, *D. cernua*, *D. hispida*, *D. lyallii* and *D. ligustrifolia* seem to be non-monophyletic, and possibly *D. pubescens* and *D. aurantiaca* too although the results are poorly resolved (Fig. 6). The delimitations of all these taxa are probably in need of revision.

More research is needed, however. We were unable to test the monophyly of a number of species (*Payera decaryi*, *P.* ‘*vondrozoensis*’, *P.* ‘*vangaindranoensis*’, *P. glabrifolia*, *Schismatoclada* ‘*papangoensis*’, *S. mandrarensis*, *S.* ‘*andringitrensis*’, *S. bracteata*, *S.* ‘*binara*’, *S.* ‘*avatara*’ and *S.* ‘*ambalabeensis*’, and *Danais volubilis*, *D. comorensis*, *D. verticillata*, *D.* ‘*andohahelensis*’, *D.* ‘*pedunculata*’, *D.* ‘*ranomafanensis*’, *D.* ‘*anjozorobeensis*’, *D. baronii* Homolle and *D.* ‘*manjakatompensis*’), since we could only sample one individual for each of these species. The monophyly of *S.* ‘*anjanaharibeensis*’ (northern clade 137) is neither rejected nor supported by our results.

### Geographically driven evolution of Danaideae

Our results show that there is a strong association between geography and evolutionary relationships in Danaideae. In all three genera, we recover many geographically segregated lineages. Species from the same geographical area tend to be more closely related to each other than they are to species from other more distant areas. The predominantly (sub)montane habitats of most Danaideae seem to be the driving factor, explaining the importance of geographical isolation in their evolution and the resulting patterns of endemism. Furthermore, the observed pattern also appears to fit the general pattern that rare, localized species (or narrowly endemic species) generally maintain the geographical signal of their diversification (e.g. Abellán and Ribera, 2017). Almost all species of *Payera* and *Schismatoclada* are narrow endemics (Buchner and Puff, 1993; this study). This geographical phylogenetic clustering indicates that the species of Danaideae seem to be limited in their dispersal ability, which is surprising given that all species of Danaideae produce capsular fruits and wind-dispersed seeds. In other words, their winged seeds may not be easily dispersed by wind compared to winged seeds of plants growing in open habitats. On the other hand, the restricted distributions of most Danaideae species might partly be related to limitations of their seed germination and/or seedling growth and survival in different environmental conditions.

#### *Evidence of a southeast-tip origin and parallel northward diversification of* Payera *and* Schismatoclada.

Despite their disparity in species richness, *Payera* and *Schismatoclada* display an apparent congruence in geographical distribution and diversification patterns, with the southeastern-tip clade and south(-central)eastern clades forming basal grades in both genera (Fig. 3). Interpreting the phylogenetic pattern, it is possible that the lowland rainforests and littoral forests of the southeast tip of Madagascar is the area of origin of both *Payera* and *Schismatoclada*, with a putative northward diversification. Few previous studies have been able to disclose potential geographical areas of origin and species diversification on Madagascar more precisely, in particular for plants, but this conclusion stands in contrast to the suggested northern origin of some Malagasy animal groups (e.g. Boumans *et al.*, 2007; Gehring *et al.*, 2013). It is also unexpected given that the southeast tip of Madagascar is the most isolated part of the island. Future biogeographical work may provide clarity.

### Geography better predictor of evolutionary relationships than morphology

In contrast to geographical proximity, gross morphology (namely leaf, stipule and flower features) appears to have little phylogenetic value in Danaideae. Our results demonstrate notable parallelism in various morphological traits traditionally used in the taxonomic delimitations of *Payera* and *Schismatoclada*. For example, species with relatively small leaves (*S.* ‘*ambohivohitrensis*’, *S.* ‘*andringitrensis*’, *S.* ‘*binara*’, *S.* ‘*didyensis*’, *S.* ‘*gracilis*’, *S. marojejyensis*, *S.* ‘*keraudreniana*’, *S.* ‘*papangoensis*’ and *S. spathulata*) belong to at least six lineages within *Schismatoclada*, and species with small and spathulate leaves (*S.* ‘*andringitrensis*’, *S.* ‘*gracilis*’, *S.* ‘*keraudreniana*’ and *S. spathulata*) are nested in four distinct lineages (Fig. 5). Remarkable parallelism has also been reported in the following morphological features of other Rubiaceae lineages: calycophylls, breeding systems and locule number (e.g. tribe Vanguerieae, Razafimandimbison *et al.*, 2009*b*; tribe Condamineeae, Kainulainen *et al.*, 2010); inflorescence type (e.g. tribe Morindeae, Razafimandimbison *et al.*, 2009*a*); and fruit size (e.g. Lantz and Bremer, 2004) and dehiscence (e.g. Kainulainen *et al.*, 2010).

## CONCLUSIONS

The monophyly of the tribe Danaideae and its three genera (*Danais*, *Payera* and *Schismatoclada*) is supported. Numerous geographically separated but morphologically heterogeneous lineages and a high level of previously undocumented morphological and species diversity of Danaideae on Madagascar are revealed. This will result in two-fold and one-third increases of *Schismatoclada* and *Payera* species, respectively. Geographical proximity (i.e. geography) is in general better than morphology-based taxonomy in predicting evolutionary history (i.e. phylogeny) in Danaideae.

Systematists may often prefer to conduct molecular phylogenetic studies on groups of organisms that have been revised, taxonomically. Here, we have adopted a novel approach by performing rigorous molecular phylogenetic analyses of Danaideae based on a relatively dense taxon sampling, as a precursor to taxonomic revisions of its genera (*Payera* and *Schismatoclada*), classically achieved using morphological similarities and/or dissimilarities to delineate taxa. This approach enables integration of molecular and morphological evidence more efficiently during the process of taxonomic revisions. It allows us to test the monophyly of as yet undescribed taxa proposed based on morphology, using molecular data, and reveals their phylogenetic placements prior to the publication of the revisions. Such crucial information can thus be a part of the original decision of whether a species should be described as new. Furthermore, the molecular evidence for the monophyly of rare, new, undescribed species is a strong justification for formally describing such species even based on incomplete material. Finally, our approach allows systematists to formulate a more accurate species diagnosis, traditionally based solely on morphological similarities.

## SUPPLEMENTARY DATA

Supplementary data are available online at https://academic.oup.com/aob and consist of the following. Figure S1: Bayesian majority-rule consensus trees based on non-clock analyses of single gene regions. Table S1: List of Danaideae species accepted in the present study, and provisionally proposed new species. Table S2: List of investigated plant material, voucher information, country origins and GenBank accession numbers. Table S3: Results from the individual relaxed-clock analyses, and from combining these distributions into one. File S1: The combined data matrix in nexus format.

mcac121_suppl_Supplementary_Figures

mcac121_suppl_Supplementary_Table_S1

mcac121_suppl_Supplementary_Table_S2

mcac121_suppl_Supplementary_Table_S3
