# Supplementary figures and images for: Phylogeny of the Madagascar-centred tribe Danaideae (Rubiaceae) as a precursor to taxonomic revision: insights into its generic and species limits, affinities and distribution
Source: Ann Bot. 2022 Sep 23;130(6):849–67. doi: 10.1093/aob/mcac121 (PMC9758302; doi:10.1093/aob/mcac121)

## Slide 1
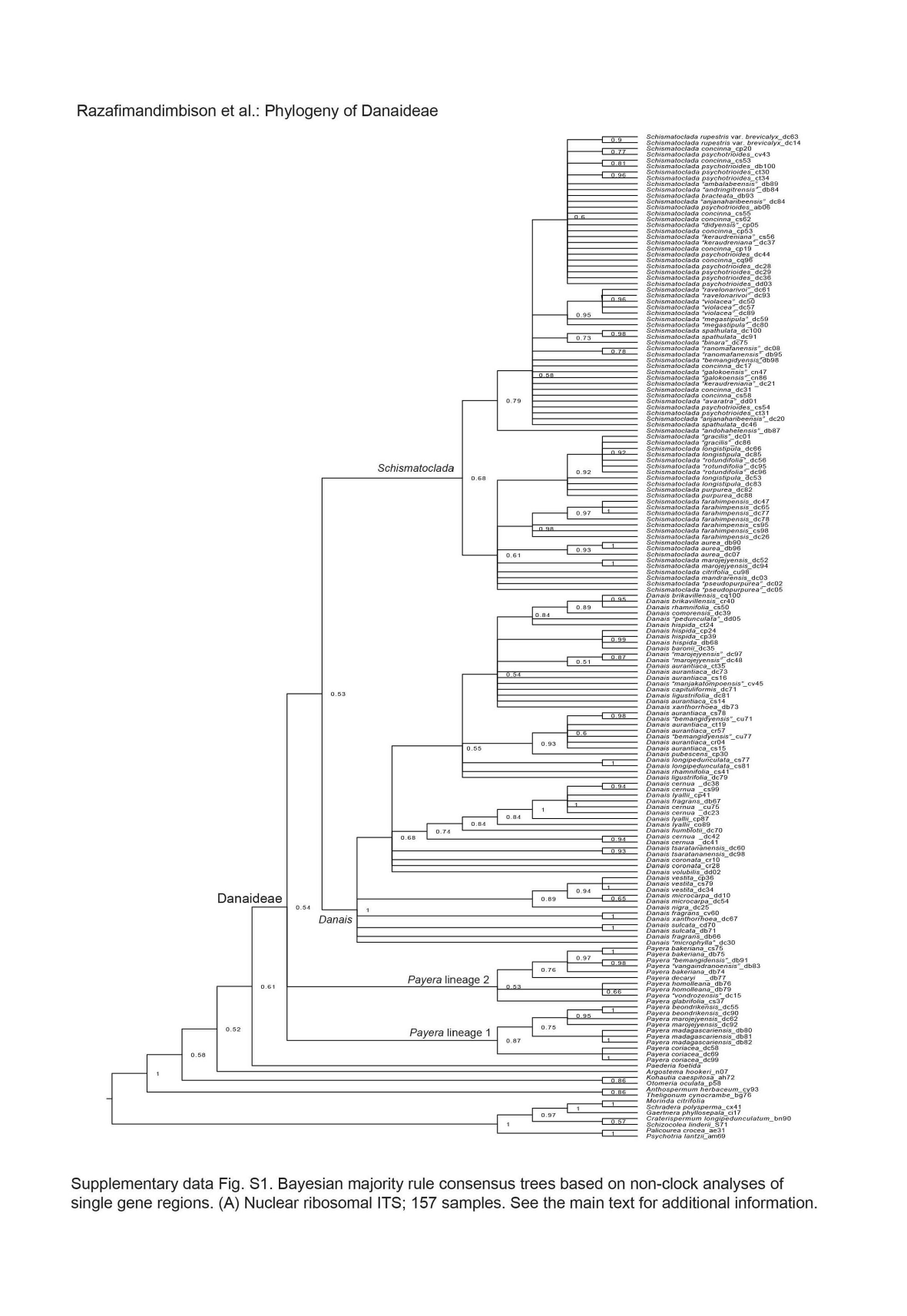

## Slide 2
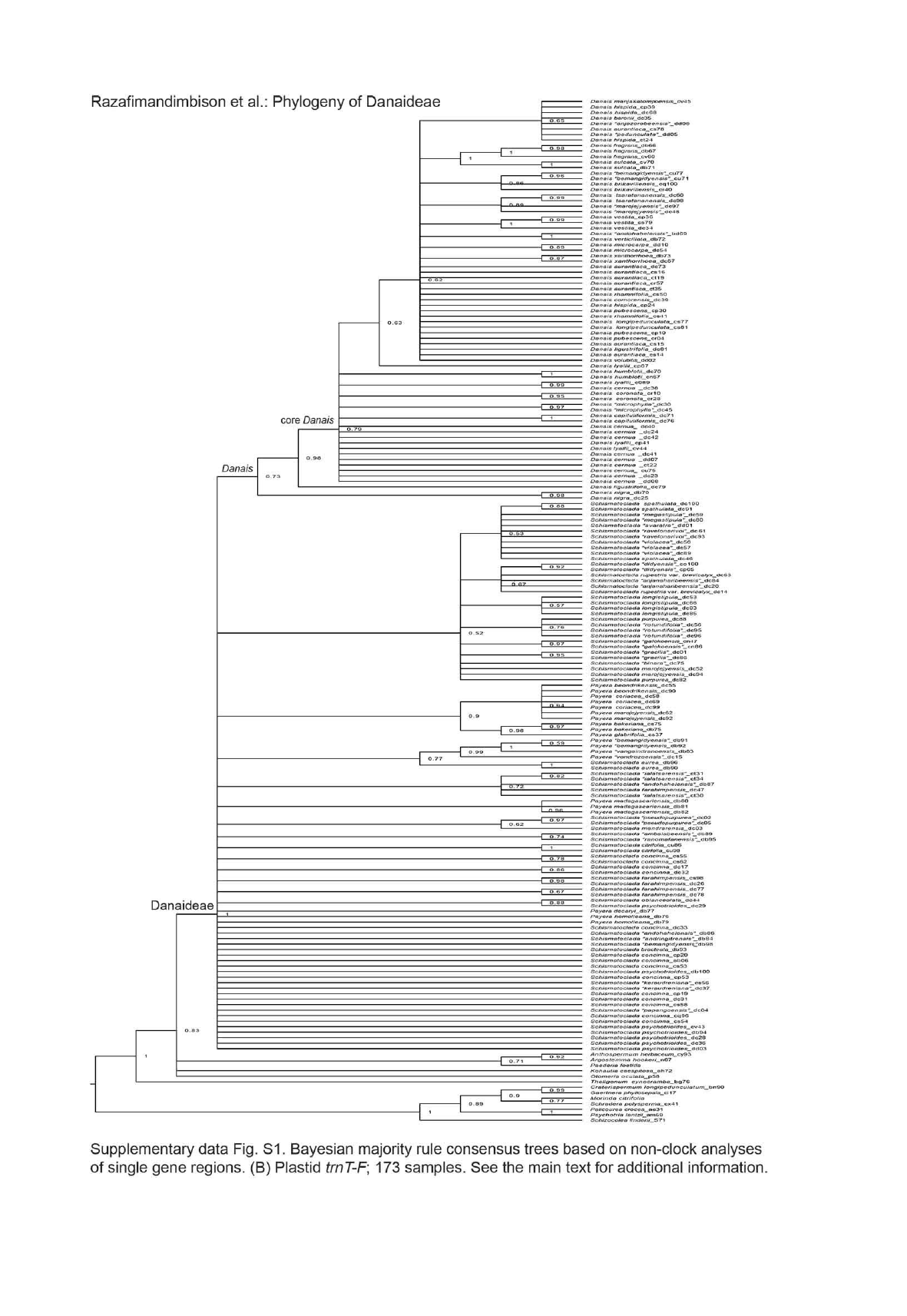

## Slide 3
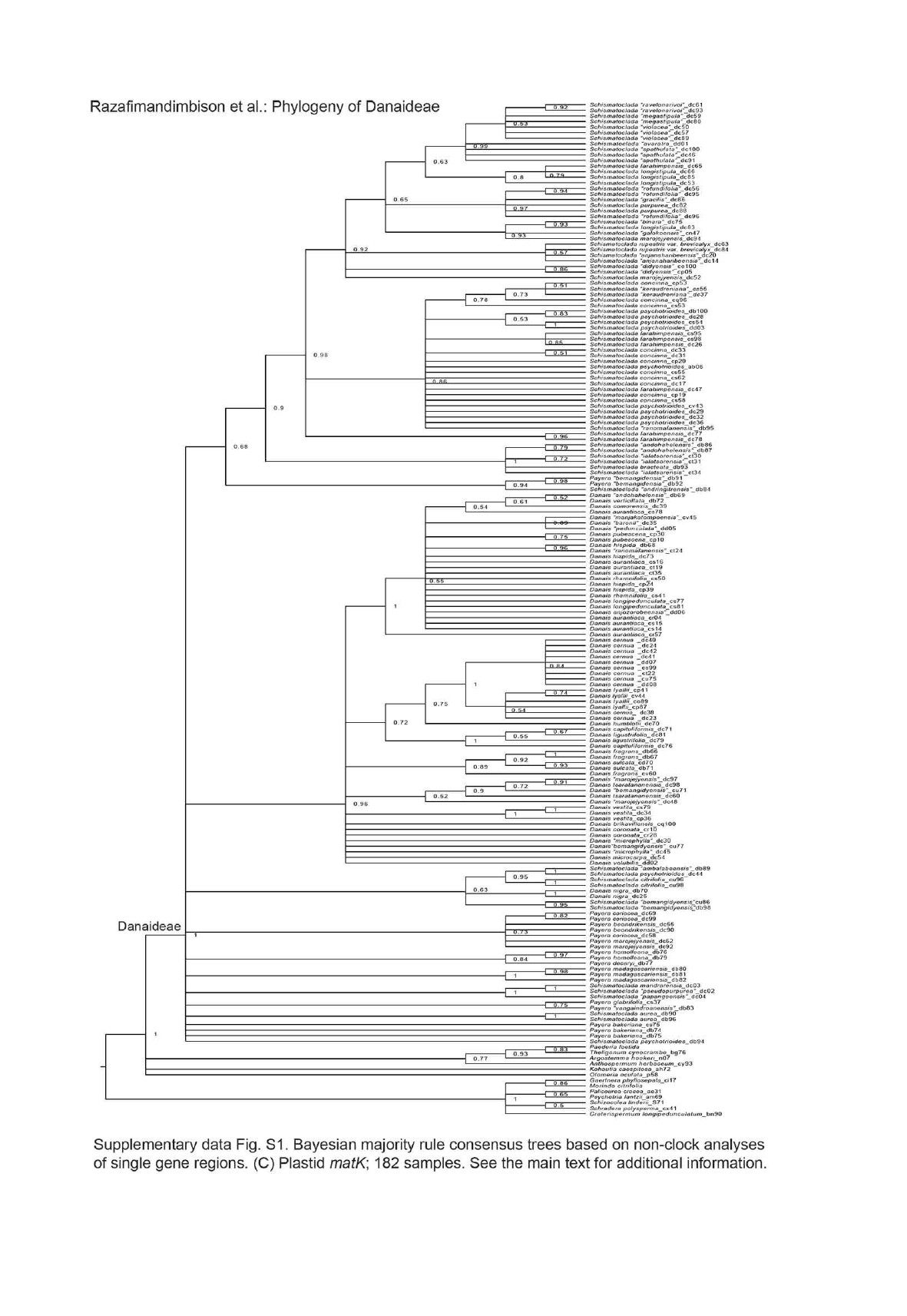

## Slide 4
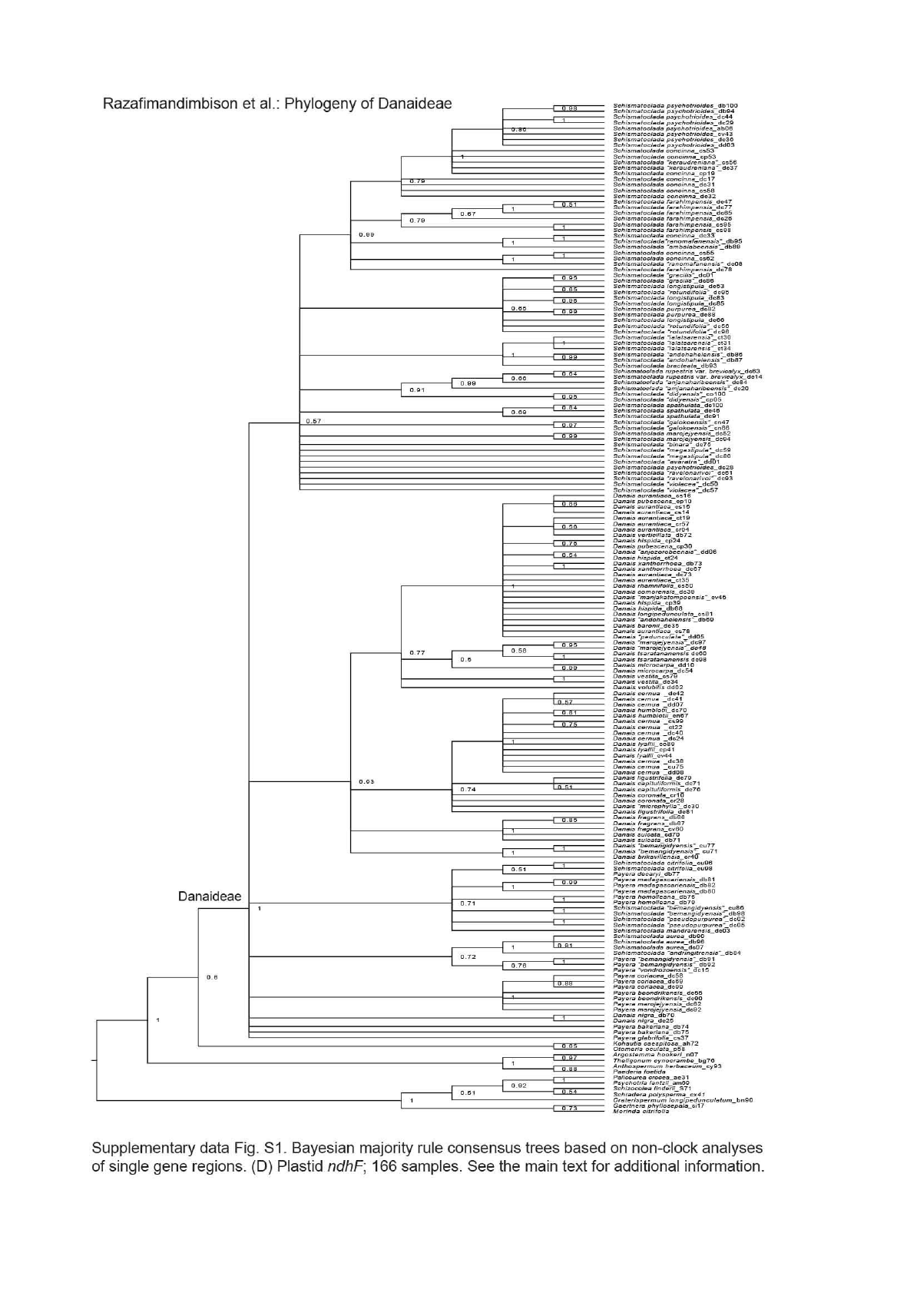

Supplement: mcac121_suppl_Supplementary_Figures [file mcac121_suppl_supplementary_figures.pptx]
